# A murine model of the human CREBRF^R457Q^ obesity-risk variant does not influence energy or glucose homeostasis in response to nutritional stress

**DOI:** 10.1371/journal.pone.0251895

**Published:** 2021-09-14

**Authors:** Jitendra S. Kanshana, Polly E. Mattila, Michael C. Ewing, Ashlee N. Wood, Gabriele Schoiswohl, Anna C. Meyer, Aneta Kowalski, Samantha L. Rosenthal, Sebastien Gingras, Brett A. Kaufman, Ray Lu, Daniel E. Weeks, Stephen T. McGarvey, Ryan L. Minster, Nicola L. Hawley, Erin E. Kershaw

**Affiliations:** 1 Division of Endocrinology and Metabolism, Department of Medicine, University of Pittsburgh, Pittsburgh, PA, United States of America; 2 Department of Pharmacology and Toxicology, University of Graz, Graz, Austria; 3 Center for Craniofacial and Dental Genetics, Department of Oral and Craniofacial Sciences, University of Pittsburgh, Pittsburgh, PA, United States of America; 4 Department of Immunology, University of Pittsburgh, Pittsburgh, PA, United States of America; 5 Division of Cardiology, Department of Medicine, University of Pittsburgh, Pittsburgh, PA, United States of America; 6 Department of Molecular and Cellular Biology, College of Biological Science, University of Guelph, Guelph, ON, Canada; 7 Department of Human Genetics, Graduate School of Public Health, University of Pittsburgh, Pittsburgh, Pennsylvania, United States of America; 8 Department of Biostatistics, Graduate School of Public Health, University of Pittsburgh, Pittsburgh, Pennsylvania, United States of America; 9 International Health Institute, Department of Epidemiology, Brown University School of Public Health, Providence, Rhode Island, United States of America; 10 Department of Anthropology, Brown University, Providence, Rhode Island, United States of America; 11 Department of Chronic Disease Epidemiology, Yale School of Public Health, New Haven, Connecticut, United States of America; Max Delbruck Centrum fur Molekulare Medizin Berlin Buch, GERMANY

## Abstract

Obesity and diabetes have strong heritable components, yet the genetic contributions to these diseases remain largely unexplained. In humans, a missense variant in Creb3 regulatory factor (CREBRF) [rs373863828 (p.Arg457Gln); CREBRF^R457Q^] is strongly associated with increased odds of obesity but decreased odds of diabetes. Although virtually nothing is known about CREBRF’s mechanism of action, emerging evidence implicates it in the adaptive transcriptional response to nutritional stress downstream of TORC1. The objectives of this study were to generate a murine model with knockin of the orthologous variant in mice (CREBRF^R458Q^) and to test the hypothesis that this CREBRF variant promotes obesity and protects against diabetes by regulating energy and glucose homeostasis downstream of TORC1. To test this hypothesis, we performed extensive phenotypic analysis of CREBRF^R458Q^ knockin mice at baseline and in response to acute (fasting/refeeding), chronic (low- and high-fat diet feeding), and extreme (prolonged fasting) nutritional stress as well as with pharmacological TORC1 inhibition, and aging to 52 weeks. The results demonstrate that the murine CREBRF^R458Q^ model of the human CREBRF^R457Q^ variant does not influence energy/glucose homeostasis in response to these interventions, with the exception of possible greater loss of fat relative to lean mass with age. Alternative preclinical models and/or studies in humans will be required to decipher the mechanisms linking this variant to human health and disease.

## Introduction

Obesity is a global public health threat that is associated with additional metabolic abnormalities (i.e., insulin resistance, dyslipidemia) that increase the risk of common diseases such as diabetes and cardiovascular disease. Although obesity clearly has a complex and multifactorial etiology, it has a strong heritable component (>80% in some studies). Over the past several decades, large genome-wide association studies (GWAS) have sought to identify the genetic contributions to this heritability. These studies have been instrumental in linking numerous genes/loci to anthropometric and/or metabolic traits, including body mass index (BMI)/obesity [1, 2], regional adiposity/insulin biology [2, 3], and glycemia/diabetes [4]. These studies have revealed important insights into the underlying physiology/pathophysiology of these traits such as the critical role of central neuroendocrine regulation of energy homeostasis in obesity [1, 2], adipose tissue biology in insulin resistance [2, 3], and beta cell biology in diabetes [4]. Despite these insights, the majority of heritability of these metabolic traits remains largely unexplained. For example, genes/loci identified by GWAS account for less than 3% of the variance in BMI [1]. Furthermore, these GWAS are historically enriched in populations with European ancestry and have not adequately assessed genetic contributions from underrepresented groups [5], many of which are disproportionately affected by obesity, diabetes, and cardiometabolic diseases. Identifying novel genes/pathways underlying this “missing heritability” across diverse populations could improve prevention and/or treatment of obesity and its complications.

To bridge this knowledge gap, our research group has established an ongoing collaboration with the Samoan Ministry of Health to understand and address cardiometabolic disease risk in Samoans, a geographically isolated population with a particularly high prevalence of obesity. This collaborative effort resulted in the identification of a missense variant in Creb3 regulatory factor (CREBRF) [rs373863828 (p.Arg457Gln); CREBRF^R457Q^] by GWAS in Samoans that was strongly associated with BMI [6]. This obesity-risk variant (odds ratio for obesity of 1.305 per copy) is unique in that, even though it has a BMI-increasing effect size greater than any other known common obesity-risk variant (1.36–1.45 kg/m^2^ CREBRF^R457Q^ versus only 0.37 kg/m^2^ for the FTO locus), it is paradoxically associated with relative protection from diabetes (odds ratio for diabetes of 0.586 after controlling for BMI) and other metabolic complications of obesity [7]. This variant is also positively related to linear height in humans [8–10]. Although this variant is extremely rare in populations most commonly studied in GWAS (i.e., Europeans), it is very common in Samoans (~46% carry at least one copy of the risk allele). The relationship between CREBRF^R457Q^ and obesity/diabetes risk has now been replicated by numerous other groups in Oceanic populations [9, 11–15]. However, despite its large effect size and high prevalence in this population, the mechanisms by which the CREBRF^R457Q^ variant contributes to obesity and obesity-associated traits remain unknown.

CREBRF (also known as Luman/CREB3 Recruitment Factor, LRF) was initially identified in 2008 as a novel protein that binds to CREB3 and inhibits CREB3-mediated activation of the unfolded protein response (UPR) in mice [16]. Global targeted deletion of CREBRF in mice results in lower total body mass [17], suggesting that the human CREBRF^R457Q^ variant may be a gain-of-function variant with respect to body mass / energy homeostasis. This hypothesis is further supported by our data demonstrating that ectopic overexpression of the human CREBRF^R457Q^ variant in a murine 3T3-L1 adipocyte model promotes adipogenesis, decreases mitochondrial respiration, and increases fat storage [6]. While these data implicate adipocytes in the pathophysiology of the CREBRF^R457Q^ variant, the relative contributions of other tissues and cell types where CREBRF is also expressed remain unknown [6]. CREBRF is highly regulated by nutritional status and TORC1 action (increased by fasting/starvation and TORC1 inhibition; decreased by refeeding and TORC1 activation) [6, 18]. Overexpression of CREBRF in 3T3-L1 adipocytes protects against starvation [6], whereas deletion of CREBRF in Drosophila S6 cells, larvae, and adults increases susceptibility to starvation [18]. These data suggest that CREBRF plays a critical role in the cellular and physiological response to nutritional stress. In addition, mechanistic studies in Drosophila demonstrate that CREBRF mediates the majority of the downstream effects of TORC1 inhibition by rapamycin [18]. Although similar findings have yet to be confirmed in mammals, these data nonetheless implicate CREBRF in one of the most well-known energy sensing pathways in biology.

Despite the potential importance of these findings, virtually nothing is known about the function or physiological relevance of CREBRF or the CREBRF^R457Q^ variant. Our overall goal is to understand how the CREBRF^R457Q^ variant influences energy and metabolic homeostasis. Based on the above data, we hypothesized that the CREBRF^R457Q^ variant promotes obesity and protects against diabetes by regulating gene pathways involved in energy and glucose homeostasis downstream of TORC1. To test this hypothesis, we generated an animal model of the human CREBRF^R457Q^ variant by using CRISPR-Cas9 gene-editing technology to knockin the orthologous mutation in mice (CREBRF^R458Q^). We then phenotyped these mice at baseline and in response to acute (fasting/refeeding), chronic (low- and high-fat diet feeding), and extreme (prolonged fasting) nutritional stress as well as with pharmacological TORC1 inhibition and aging to 52 weeks.

## Materials and methods

### Generation and validation of *Crebrf* knockin (*Crebrf*KI) mice

**Fig 1:**
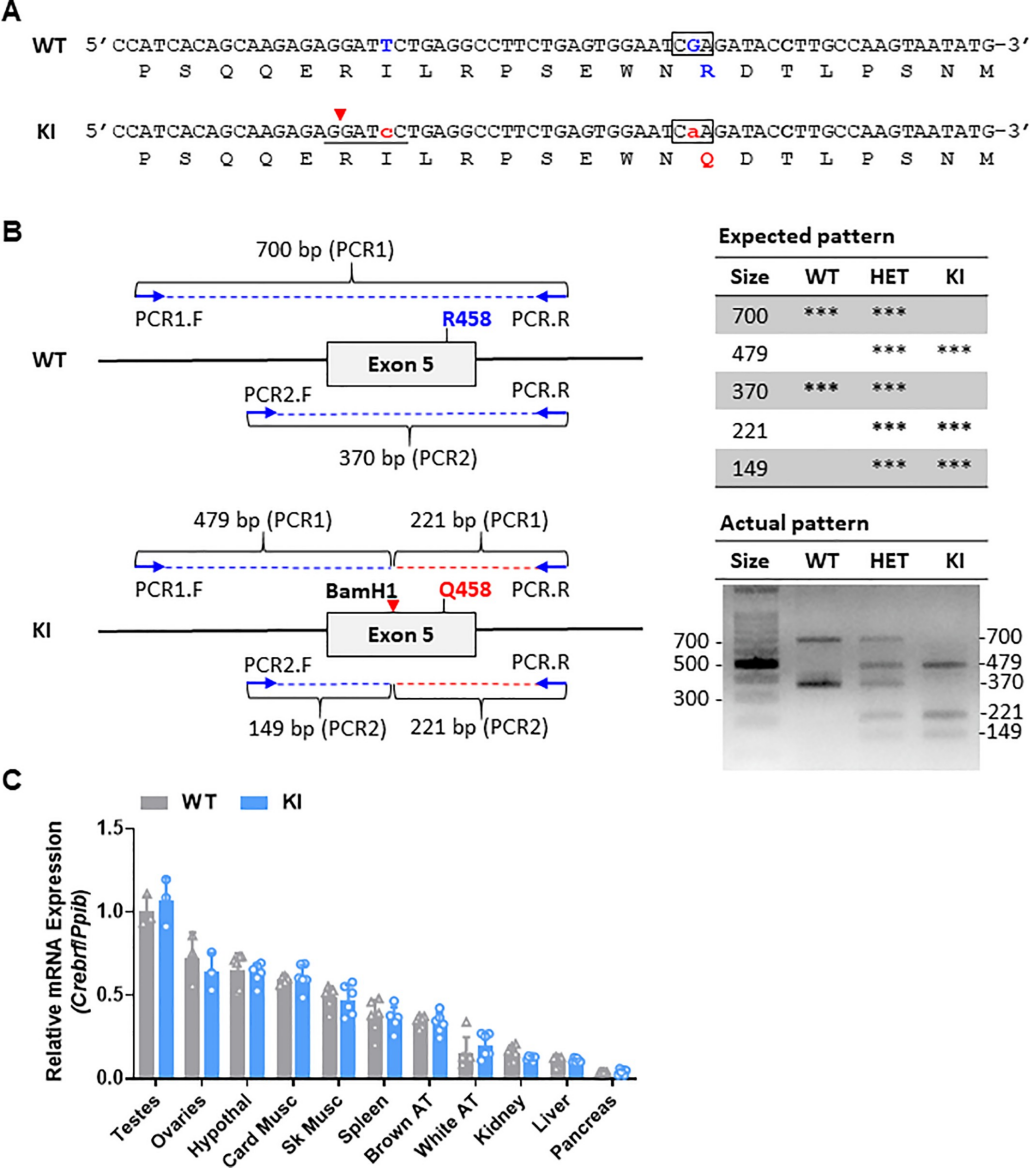
Generation and validation of *Crebrf* knockin (*Crebrf*KI) mice. **(A)** The CREBRF^R458Q^ knockin allele was generate using CRISPR-Cas9 gene-editing technology. Genomic (top) and protein (bottom) sequence of the wild-type (WT) and gene-edited knockin (KI) alleles are shown. To generate the KI allele, two substitutions were introduced: i) a silent substitution (TTC to TcC) to introduce a BamH1 site (underlined with restriction cut site shown with red arrowhead) about 20 bp away from R458 codon, to serve as a proxy for the mutation for genotyping purpose; and ii) a coding substitution (CGA to CaA) to introduce the desired R458Q knockin variant (surrounded by the black box); **(B)** The WT and KI alleles were confirmed by direct sequencing and restriction length polymorphism (RFLP) analysis. The schematic for the two-step nested PCR RFLP assay is shown on the left, and the resulting representative gels, confirming homozygous WT, heterozygous (Het), and homozygous KI mice, are shown on the right. **(C)** *Crebrf* mRNA expression by qPCR in multiple tissues of WT and KI mice (M and F, 6-week-old, ad lib fed LFD, n = 6/group except testes and ovaries n = 3/group). Data are murine *Crebrf* relative to reference gene *Ppib* and expressed relative to WT testes. Testes, ovaries, hypothalamus (hypothal), cardiac muscle (Card Musc), gastrocnemius-plantaris-soleus skeletal muscle (Sk Musc), spleen, brown adipose tissue (brown AT), perigonadal white adipose tissue (white AT), liver, and pancreas show no genotype effects on Crebrf mRNA expression. Unpaired two-tailed t-tests were used to compare gene expression (dependent variable) between genotype (fixed factor). Data are mean (SD). p<0.05 was considered significant.

CREBRF^R458Q^ knockin (*Crebrf*KI) mice were generated using CRISPR-Cas9 gene-editing technology in a congenic C57BL/6J mouse background strain with the assistance of the University of Pittsburgh Transgenic and Gene Targeting (TGT) Core [19–21]. Briefly, a double-strand DNA break was introduced in proximity of the murine R458 codon by targeting the Cas9 nuclease with a small guide RNA (sgRNA) (murine *Crebrf* target sequence: 5’-CTGGTACATATTACTTGGCA-3’, chr17:26,757,961–26,757,980 (mm10)). The desired genomic DNA substitutions were introduced by co-injection of a PAGE-purified single stranded oligodeoxynucleotide (ssODN) ultramer to serve as template for homology-directed repair (HDR). Two substitutions (lower cap bold in the sequence below; see also **Fig 1A**) were introduced: i) a silent substitution (TTC to TcC) to introduce a BamH1 site (underlined) approximately 20 bp away from the R458 codon, to serve as a proxy for the mutation for genotyping purposes; and ii) a coding substitution (CGA to CaA) to introduce the desired R458Q knockin variant. A PCR-generated sgRNA template was used for sgRNA synthesis using the MEGAshortscript T7 Kit (ThermoFisher Scientific), and Cas9 mRNA was produced using a linearized plasmid as template for the in vitro transcription mMESSAGE mMACHINE T7 Ultra Kit (ThermoFisher Scientific). Both sgRNA and Cas9 mRNA were purified using the MEGAclear Kit (ThermoFisher Scientific). C57BL/6J pronuclear-stage zygotes, obtained by natural mating of superovulated females, were microinjected with sgRNA (10 ng/μL, each) and Cas9 mRNA (20 ng/μL), and the ssODN (1μM). Injected zygotes were cultured overnight and 2-cell embryos were transferred to pseudopregnant CD1 mice to obtain potential founder mice.

The presence or absence of the desired knockin allele was confirmed in several ways. First, purified tail DNA from potential founders were screened using a two-step nested PCR-RFLP assay. The first amplification (with genomic DNA as template) used forward primer 5’ TTTAATGCCTGGCACCATTT and reverse primer 5’ GAACGAGGCAGAGGATTCAA to generate a 700 bp product (PCR product 1, PCR1). The second amplification (with PCR product 1 as template) used forward primer 5’ TGACAATTGTGGGACCATGT and reverse primer 5’ GAACGAGGCAGAGGATTCAA to generate a 370 bp product (PCR product 2, PCR2). Both steps used PCR conditions of 95°C x 30s / 55°C x 30s / 72°C x 30s for 35 cycles. The second PCR product was then digested with BamH1 and resolved on agarose, resulting in 370 bp (plus 700 bp) bands for the wild-type (WT) allele and a 221 bp and 149 bp (plus 479 bp) bands for the knockin (KI) allele (**Fig 1B**). Second, for founders positive for the KI allele by the PCR-RFLP assay, the 700 bp PCR products were subcloned into the pCR™4-TOPO^®^ TA vector and transfected into *Escherichia coli* DH5α cells (TOPO 4 Kit, ThermoFisher Scientific) followed by plasmid preparation and direct Sanger sequencing. In this way, a founder carrying the desired KI allele was confirmed and then mated to WT C57BL/6J mice to expand the colony. Subsequently, mice were genotyped using our previously published locked nucleic acid assays [21]. All oligonucleotide sequences used in this study are listed in **S1 Table**.

### Animals

Mice were housed under standard conditions (25°C, 14:10 h light:dark cycle) with *ad libitum* access to a control low-fat diet (LFD: Research Diets D12450Hi; 10/70/20 kcal% fat/carbohydrate/protein; 3.82 kcal/g) or high-fat diet (HFD: Research Diets D12451i; 45/35/20 kcal% fat/carbohydrate/protein; 4.73 kcal/g) from weaning until sacrifice, except as otherwise noted (i.e., during fasting). The LFD was specifically formulated as the control for the HFD. For the longitudinal comprehensive phenotyping experiment, male and female WT and KI mice were fed LFD or HFD until 22 weeks of age and then sacrificed in fasted (16h) or refed (24h of fasting followed by 16h of refeeding) states. Additional mice were generated to determine the effects of genotype in the context of more extreme physiological stress (prolonged fasting, age) and pharmacological stress (mTORC1 inhibition). Females were allocated to the former because metabolic phenotypes are less affected by multi-animal housing during prolonged experiments, and female mice can be housed five per cage. For the prolonged fasting experiment, female WT and KI mice were ad lib fed HFD as above. At 36 weeks of age, mice were acclimatized to single housing in metabolic cages (Sable Promethion system), fasted until they lost 25% of their initial body weight and then refed until they re-established their initial body weight. For in vivo mTORC1 inhibition experiments, male WT and KI mice were ad lib fed LFD or HFD as above, but were treated with either vehicle (Veh: sterile 10% PEG 400/8% ethanol plus an equal volume of 10% Tween 80) or rapamycin (Rapa: rapamycin 4 mg/kg body weight dissolved in the above vehicle; rapamycin was obtained LC Laboratories) via an intraperitoneal (ip) injection every other day for three weeks prior to sacrifice in the ad lib fed state at 22 weeks of age based on a previously reported protocol [22]. For the aging experiment, HFD-fed mice female mice were aged until 52 weeks. Animal experiments were approved by the University of Pittsburgh IACUC (Protocol #20107971) and conducted in conformity with PHS Policy for Care and Use of Laboratory Animals (PHS Assurance #D16-00118).

### Metabolic and biochemical analyses

Body composition, energy expenditure, and metabolic parameters were performed as described [23–25]. Body composition was determined by echo magnetic resonance imaging (Echo Medical Systems). Energy intake and expenditure, energy substrate utilization, and activity were determined by indirect calorimetry (Promethion Metabolic Phenotyping Cages from Sable Systems International). Plasma glucose was measured using a One-Touch FastTake glucometer (Lifescan). For glucose tolerance tests (GTTs), mice were injected ip with 1.5 g/kg glucose following a 6-h fast. Other serum parameters were determined using the following kits: insulin (Ultra Sensitive Mouse Insulin ELISA kit; Crystal Chem), triacylglycerol or TAG (Pointe Scientific Triglycerides Liquid Reagents; Fisher Scientific); non-esterified fatty acids or NEFAs (HR series NEFA-HR[2] Reagents; Wako Diagnostics), leptin (mouse leptin ELISA, Crystal Chem); and adiponectin (mouse adiponectin ELISA, Crystal Chem).

### Gene expression analysis

RNA was isolated using RNeasy Lipid Tissue Mini Kit with on-column DNase treatment (Qiagen) and then reverse transcribed into cDNA using qScript Supermix (Quanta Biosciences). Relative gene expression was then determined by quantitative PCR (qPCR; ABI QuantStudio3 System) using the standard curve method after amplification of cDNA with gene-specific primer-probe sets using PerfeCTa Fastmix II Fastmix (Quantabio). Taqman Gene Expression Assays were Mm01299053_m1 for *Crebrf* and Mm02342430_g1 for reference gene *Ppia* (ThermoFisher). qPCR was conducted in accordance with MIQE guidelines [26].

### Protein analysis by immunoblotting

All antibodies used in this study are listed in **S2 Table**. Immunoblotting was performed as described [27]. Briefly, tissues were homogenized in ice-cold RIPA lysis buffer (250mM Tris-HCl, pH 7.4, 750 mM NaCl, 5% Triton X-100, 2.5% sodium deoxycholate, 0.5% sodium dodecyl sulphate (SDS), 100 mM NaF, 2 mM Na_3_VO_4_, 1 mM phenylmethylsulfonyl (PMSF) and 1% cocktail protein protease and phosphatase inhibitors (Sigma), pH 8.0). Lysates were centrifuged at 14,000x *g* for 30 min at 4°C, and supernatants were stored at -80°C until analysis. Lysate protein was resolved by SDS-PAGE (Novex NuPAGE 4–12% Bis-Tris gels, Invitrogen) and transferred to polyvinylidene difluoride (PVDF) membranes (Millipore sigma). Phosphorylated and total proteins were identified by immunoblotting using the following primary antibodies: Phospho-S6 Ribosomal Protein (Ser240/244) Antibody #2215 and α-Tubulin (DM1A) Mouse mAb #3873 (Cell Signaling Technologies) at 1:1000 dilution. Immunoblots were evaluated using IRDye® 680RD donkey anti-mouse IgG and 800CW donkey anti-rabbit IgG (LI-COR) at 1:15,000 and scanned on an Odyssey Clx Imaging System (LI-COR). Fluorescent signals were quantified using Image Studio Software (LI-COR).

### Statistical analysis

Results are expressed as mean (SD). Comparisons were made using general linear models followed by determination of simple effects for pair-wise comparisons if relevant (SPSS Software v27). For repeated measurements, comparisons were made by generalized linear model with time as a within subject variable. For indirect calorimetry data, comparisons were made using generalized estimated equations with time as a within subject variable. Dependent variables and fixed effects for each analysis are indicated in the text and figure legends. Males and females were analyzed separately. For all analyses, p values of <0.05 were considered statistically significant.

## Results

### Generation and validation of *Crebrf* knockin (*Crebrf*KI) mice

Our previous study identified a nonsynonymous variant in CREBRF (rs373863828) to be strongly associated with obesity and diabetes risk in humans [6]. The variant is a guanine (G) to adenine (A) substitution that changes the amino acid at position 457 from an arginine [Arg, R] to a glutamine (Gln, Q), designated CREBRF^R457Q^ in humans (**S1A and S1B Fig**). CREBRF DNA and protein sequences are highly conserved in vertebrates. Comparison of human to mouse protein sequences revealed that the homologous amino acid in mice was at position 458 (**S1B Fig**). To generate *Crebrf*KI mice, CRISPR-Cas9 gene-editing technology was used to replace the guanine (G) to adenine (A) in genomic DNA of congenic C57BL/6J mice (comparable to the rs373863828[A] minor allele in humans), to generate the homologous mutant protein, designated CREBRF^R458Q^ in mice. The annotated murine wild-type (WT) and genome-edited knockin (KI) alleles (**Fig 1A**) were confirmed by direct sequencing and restriction fragment length polymorphisms (RFLP) (**Fig 1B**). Resulting mice homozygous for the KI allele are subsequently designated *Crebrf*KI or KI mice. Gene expression analysis confirmed *Crebrf* mRNA expression across multiple tissues in WT and KI mice, and this expression did not differ between genotypes (**Fig 1C**). Endogenous CREBRF protein expression is known to be low and transiently expressed at baseline [16] and could not detected in peripheral tissues of WT of KI mice. Experimental WT and KI mice, generated from heterozygous x heterozygous crosses, were viable and fertile with the expected Mendelian ratios of genotype and sex in resulting pups.

### The CREBRF^R458Q^ variant does not affect body weight, body composition, or linear growth at baseline (low-fat diet) or in response to chronic nutritional overload (high-fat diet) in mice

**Fig 2:**
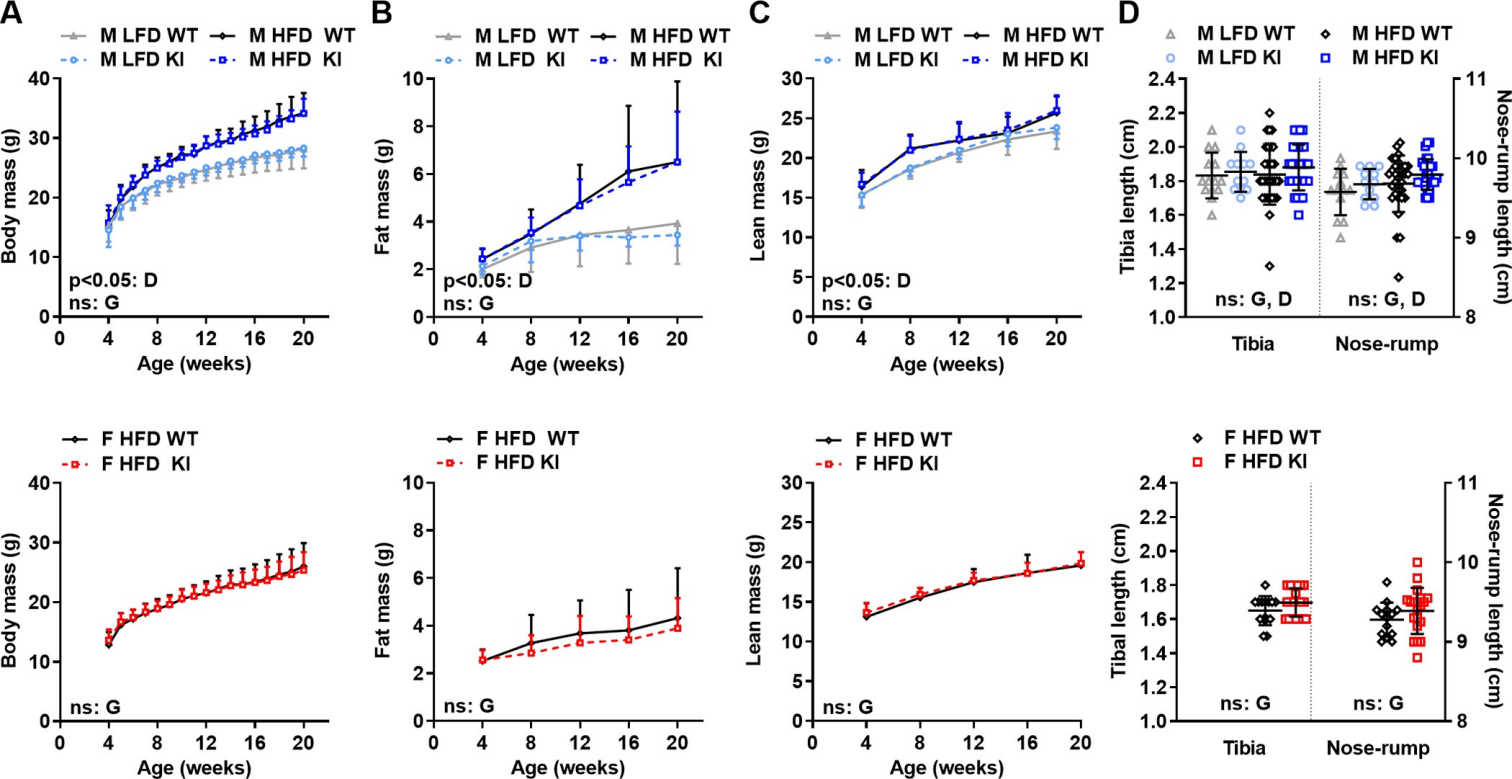
Effect of the CREBRF^R458Q^ variant on body weight, body composition, and linear growth on control diet (low-fat diet) and in response to chronic nutritional overload (high-fat diet) in mice. Male (M, top row, n = 12-14/group for LFD and 18/group for HFD) and female (F, bottom row, n = 30-42/group) wild-type (WT) and knockin (KI) mice were fed low-fat diet (LFD) or high-fat diet (HFD) weaning until sacrifice at 22 weeks of age (or 36 weeks of age in Fig 5). Total body mass (**A**), fat mass (**B**), and lean mass (**C**) were determined at the ages indicated. Tibia and Nose-rump length (**D**) were determined at 22 weeks of age (M, n = 12-14/group for LFD and 30-31/group for HFD; F n = 14-20/group). General linear models with (**A-C**) or without (**D**) time as a within subject variable were used to compare the above dependent variables between D = diet (LFD, HFD) and G = genotype (WT, KI) as fixed factors. Data are mean (SD). Main effects with a significance level of p<0.05 are indicated. Main effects with a significance level of ≥0.05 are labeled as non-significant (ns).

The human CREBRF^R457Q^ variant is associated with greater body mass [6, 11–13] and linear height [8–10] in humans. To determine the effect of the murine CREBRF^R458Q^ variant on these parameters, we evaluated energy homeostasis, body composition, and linear growth in male and female WT and KI mice under control nutritional conditions (low-fat diet, LFD; males only) and in response to chronic nutritional overload (high-fat diet, HFD; males and females) from weaning until sacrifice at 22 weeks of age. No differences were identified between genotypes for longitudinal total body mass (**Fig 2A**), fat mass (**Fig 2B**), or lean mass (**Fig 2C**) in male (**Fig 2, blue, top row**) or female (**Fig 2, red**, **bottom row**) mice on either diet. In addition, at the time of sacrifice, no differences were identified between genotypes for two different measures of linear growth: tibia length and nose-rump length (**Fig 2D**) or for individual tissue weights (**S2A and S2B Fig**). Notably, as positive controls, the expected effects of diet on these parameters were present, i.e., higher total body mass and fat mass with HFD-feeding (**Fig 2A and 2B top row**; **S2A Fig**). Thus, the murine CREBRF^R458Q^ variant does not affect body weight, body composition, or linear growth at baseline or in response to chronic nutritional overload in mice.

### The CREBRF^R458Q^ variant does not affect energy homeostasis in response to acute (<24h fasting/refeeding) or chronic (high-fat diet) nutritional stress in mice

**Fig 3:**
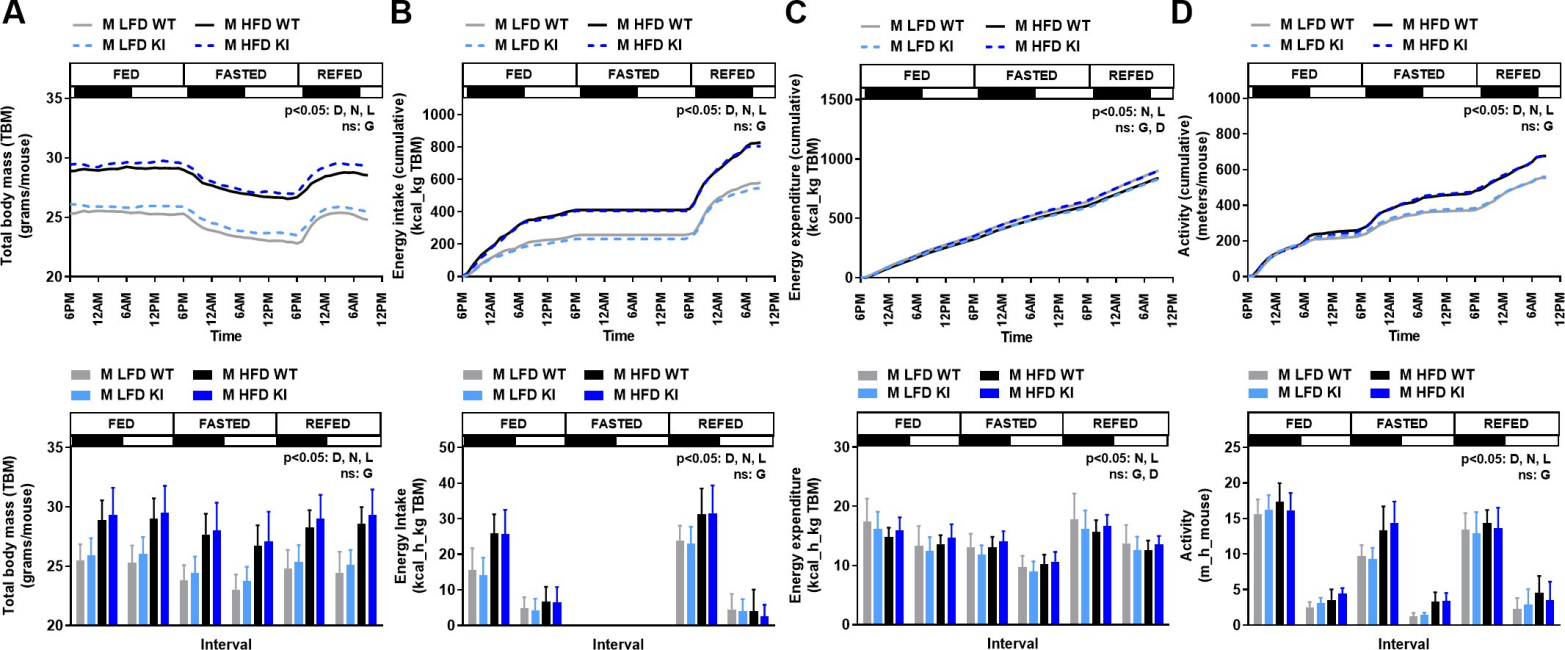
Effect of the CREBRF^R458Q^ variant on energy homeostasis in response to acute (<24h fasting/refeeding) or chronic (high-fat diet) nutritional stress in mice. Male (M, 12-week-old, n = 7-12/group) wild-type (WT) and knockin (KI) mice fed control low-fat diet (LFD) or high-fat diet (HFD) were evaluated for changes in total body mass **(A)**, energy intake **(B)**, energy expenditure **(C)**, and activity **(D)** in response to changes in nutritional status (fed, fasted, refed) over time using indirect calorimetry and activity monitors (Sable System). The protocol was as follows: 24–36 hours of acclimation, 24 hours of ad lib feeding (6PM-6PM), 24 hours of fasting (6PM-6PM), 15 hours of refeeding (6PM-9AM). Feeding status and lighting status (dark = black bar; light = white bar) are shown on the top of each graph. Data are presented in two ways: Top row = mean (without SD for figure clarity) for each variable over the course of the entire study (**A** = absolute value, **B-D** = cumulative values); or Bottom row = mean (SD) for each variable during each 12-hour light-dark interval (**A** = absolute value; **B-D** = average per hour for that interval). Generalized estimated equations with time as a within subject variable were used to compare the above dependent variables between D = diet (LFD, HFD), N = nutritional status (fed, fasted, refed), L = lighting status (dark, light), and G = genotype (WT, KI) as fixed factors. Main effects with a significance level of p<0.05 are indicated. Main effects with a significance level of ≥0.05 are labeled as non-significant (ns).

CREBRF has been shown to be highly regulated by nutritional stress in mammalian cells [6] and Drosophila [18]. Since genotype could lead to equal or conditional changes in energy intake and expenditure that do not induce a net change in body mass or composition, we additionally evaluated these parameters in WT and KI mice in response to both acute nutritional stress (<24h fasting and refeeding) and chronic nutritional stress (LFD and HFD feeding). As an initial step, we performed a detailed analysis of phenotypic parameters in association with indirect calorimetry and activity monitoring using a comprehensive laboratory animal metabolic measurement system in 12-week-old male mice fed LFD and HFD. The overall effect for each parameter is expressed as both cumulative values over time (**Fig 3, top row**) and rates per interval (light-dark intervals within fed-fasted-refed intervals) (**Fig 3, bottom row**). No differences were identified between genotypes for total body mass (**Fig 3A**), energy intake per total body mass (**Fig 3B)**, energy expenditure per total body mass (**Fig 3C**), or activity per mouse (**Fig 3A**), regardless of diet, nutritional status, or light cycle. As positive controls, the expected significant effects of diet, nutritional status, and light cycle on these parameters were present, i.e., higher total body mass, energy intake per total body mass, and activity per mouse with HFD compared to LFD, fed/refed compared to fasted states, and dark compared to light cycle; as well as higher energy expenditure per total body mass with fed/refed compared to fasted states, and dark compared to light cycle. Thus, the murine CREBRF^R458Q^ variant does not affect total body mass, energy intake, energy expenditure, or activity in response to either acute nutritional stress (fasting and refeeding) or chronic nutritional stress (LFD and HFD feeding).

### The CREBRF^R458Q^ variant does not affect energy substrate utilization, glucose homeostasis, or lipid homeostasis in response to acute (<24h fasting/refeeding) or chronic (high-fat diet) nutritional stress in mice

**Fig 4:**
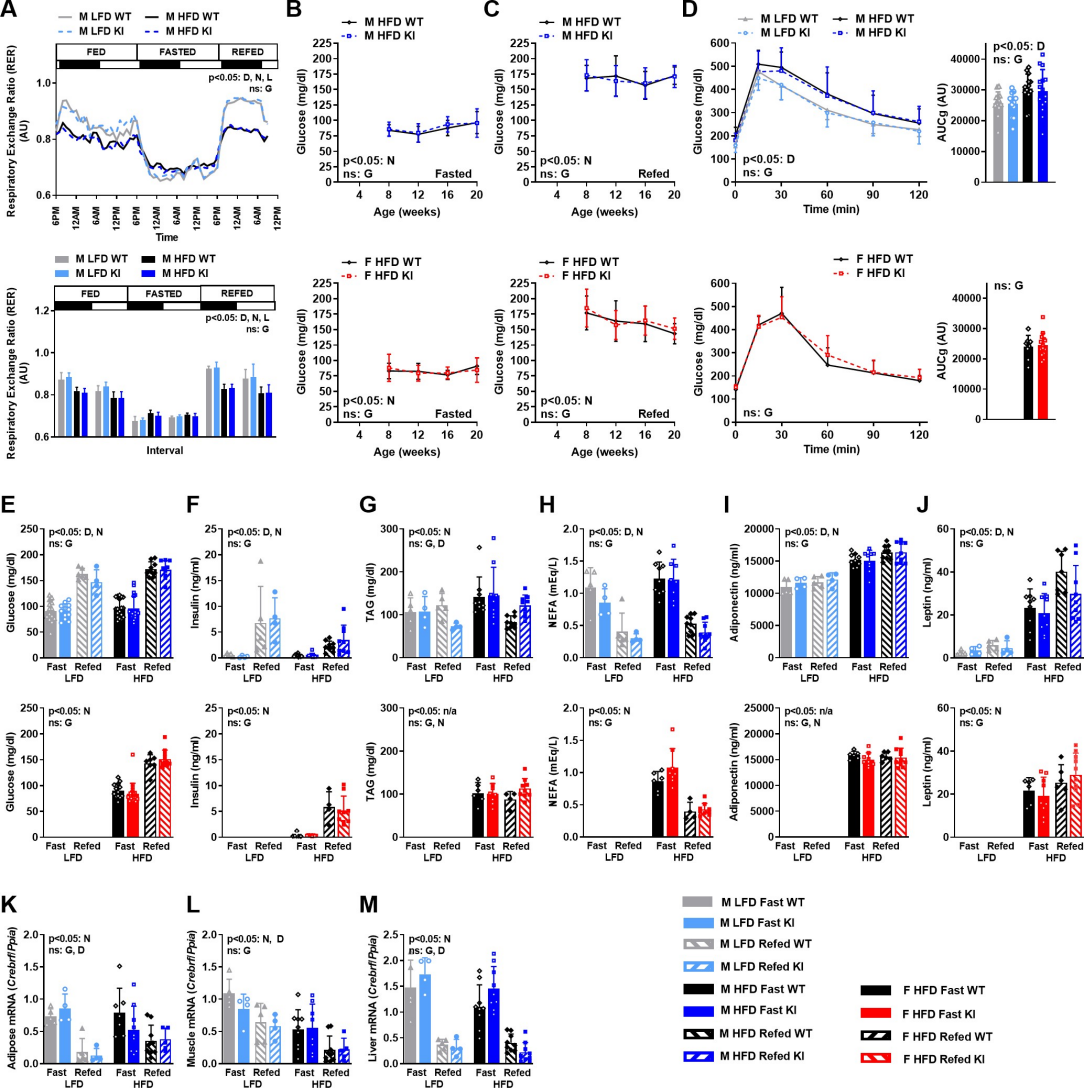
Effect of the CREBRF^R458Q^ variant on energy substrate utilization, glucose homeostasis, and lipid homeostasis in response to acute (<24h fasting/refeeding) or chronic (high-fat diet) nutritional stress in mice. Respiratory exchange ratio (RER) in male (M, 12-week-old, n = 7-12/group) wild-type (WT) and knockin (KI) mice fed low-fat diet (LFD) or high-fat diet (HFD) during changes in nutritional status (fed, fasted, refed) over time using indirect calorimetry (Sable System). The protocol, parameters, and analyses are described in the legend for **Fig 3**. **(B-C)** Blood glucose (dependent variable) at 4-week intervals from weaning until sacrifice in HFD-fed male (M, top, n = 18-19/group) and female (F, bottom, n = 14-20/group) wild-type (WT) and knockin (KI) mice in response to fasting (**B**, 16h) and refeeding (**C**, 16h fast followed by 12h refeeding). For clarity, B and C are shown as separate graphs but data are analyzed together. **(D)** Glucose tolerance tests (GTTs, left) and area under the curve for glucose (AUCg, right) in male (M, top, 19-week-old, n = 11-16/group) and female (F, bottom, 19 -week-old, n = 9-14/group) wild-type (WT) and knockin (KI) mice. **(E-J)** Serum parameters in LFD- and HFD-fed male (E-J, top) and female (E-J, bottom) WT and KI mice sacrificed at 22-weeks-old in the fasted (16h) or refed (24h of fasting followed by 16h of refeeding) states (4-9/group): **(E)** glucose, **(F)** insulin, **(G)** triacylglycerol (TAG), **(H)** non-esterified fatty acids (NEFA), **(I)** adiponectin, and **(J)** leptin. **(K-M)** Expression of *Crebrf* relative to *Ppia* mRNA in adipose tissue **(K)**, gastrocnemius skeletal muscle **(L)**, and liver **(M)** in the same mice as (E-J). General linear models with **(B-D)** or without **(E-M)** time as a within subject variable were used to compare the above dependent variables between D = diet (LFD, HFD), N = nutritional status (fasted, refed), and G = genotype (WT, KI) as fixed factors. Data are mean (SD). Main effects with a significance level of p<0.05 are indicated. Main effects with a significance level of ≥0.05 are labeled as non-significant (ns).

The human CREBRF^R457Q^ variant is associated with relative protection from metabolic complications of obesity, most notably lower fasting glucose and lower diabetes risk [6, 11–13] as well as absence of expected obesity-associated increase in serum lipids in humans [6]. To determine the effect of the murine CREBRF^R458Q^ variant on these parameters, we evaluated energy substrate utilization, glucose homeostasis, and lipid homeostasis in response to acute nutritional stress (<24h fasting and refeeding) and chronic nutritional stress (LFD and HFD feeding) in WT and KI mice. We first determined the respiratory exchange ratio (RER) using the above indirect calorimetry experiment in 12-week-old male mice fed LFD and HFD as above (**Fig 4A**). No differences were identified between genotypes for RER, regardless of diet, nutritional status, or light cycle. As positive controls, the expected significant effects of diet, nutritional status, and light cycle on these parameters were present, i.e., higher RER with LFD- compared to HFD, fed/refed compared to fasted states, and dark compare to light cycle (reflecting greater utilization of carbohydrate over lipid substrates under these conditions). Thus, the murine CREBRF^R458Q^ variant does not affect the ability to switch between glucose and lipid as energy substrates in response to acute or chronic nutritional stress. We next determined blood glucose at 4-week intervals from weaning until sacrifice in HFD-fed male (**Fig 4B and 4C, blue top**) and female (**Fig 4B and 4C, red, bottom)** mice in response to fasting (**Fig 4B**) and refeeding (**Fig 4C**). No differences were identified between genotypes for blood glucose at any time point, regardless of nutritional status. As a positive control, the expected significant effects of nutritional status on blood glucose were present, i.e., higher blood glucose in the refed compared to fasted states. We next determined the blood glucose response to an intraperitoneal glucose load using glucose tolerance tests in male (**Fig 4D, blue, top**) and female (**Fig 4D, red, bottom**) mice. No differences were identified between genotypes for glucose tolerance, regardless of diet. As a positive control, the expected significant effects of diet on glucose tolerance were present, i.e., worse glucose tolerance and greater area under the curve for glucose (AUCg) with HFD compared to LFD. The insulin secretory response following an intraperitoneal glucose challenge (1.5 g/kg) was evaluated in HFD-fed mice, but no genotype effects were identified [AUC for insulin (0-15min): males, 6/group, WT 29.64 ± 7.38 and KI 34.80 ± 13.15; females, 10-12/group, WT 17.49 ± 6.82 and KI 15.08 ± 7.25].

We additionally determined serum parameters for glucose and lipid homeostasis in LFD- and HFD-fed male (**Fig 4E–4J, blue, top**) and HFD-fed female (**Fig 4E–4J, red, bottom**) mice following sacrifice in the fasted and refed states. No differences were identified between genotypes for glucose (**Fig 4E**), insulin (**Fig 4F**), triacylglycerols (**Fig 4G**), non-esterified fatty acids (**Fig 4H**), adiponectin (**Fig 4I**), or leptin (**Fig 4J**), regardless of diet or nutritional status. As a positive control, the expected effects of diet and nutritional status on these parameters were observed, i.e., higher glucose, adiponectin, and leptin with HFD; higher glucose and insulin, lower NEFA and TAG with refeeding. Finally, we measured *Crebrf* mRNA expression and regulation by nutritional status and diet in metabolically-relevant tissues (**Fig 4K–4M**), including adipose tissue (**Fig 4K**), skeletal muscle (**Fig 4L**), and liver (**Fig 4M**). *Crebrf* mRNA was regulated by nutritional status (higher with fasting compared to refeeding) in all three tissues and by diet (higher in LFD compared to HFD) in muscle. In contrast, *Crebrf* mRNA expression did not differ by genotype in any of the three tissues. Thus, the CREBRF^R458Q^ variant does not affect energy substrate utilization, glucose homeostasis, or lipid homeostasis in response to acute (<24h fasting/refeeding) or chronic (HFD) nutritional stress in mice, despite regulation of *Crebrf* mRNA expression in response to these interventions.

### The CREBRF^R458Q^ variant does not affect energy or metabolic homeostasis in response to prolonged nutritional stress (>24h fasting) in mice

**Fig 5:**
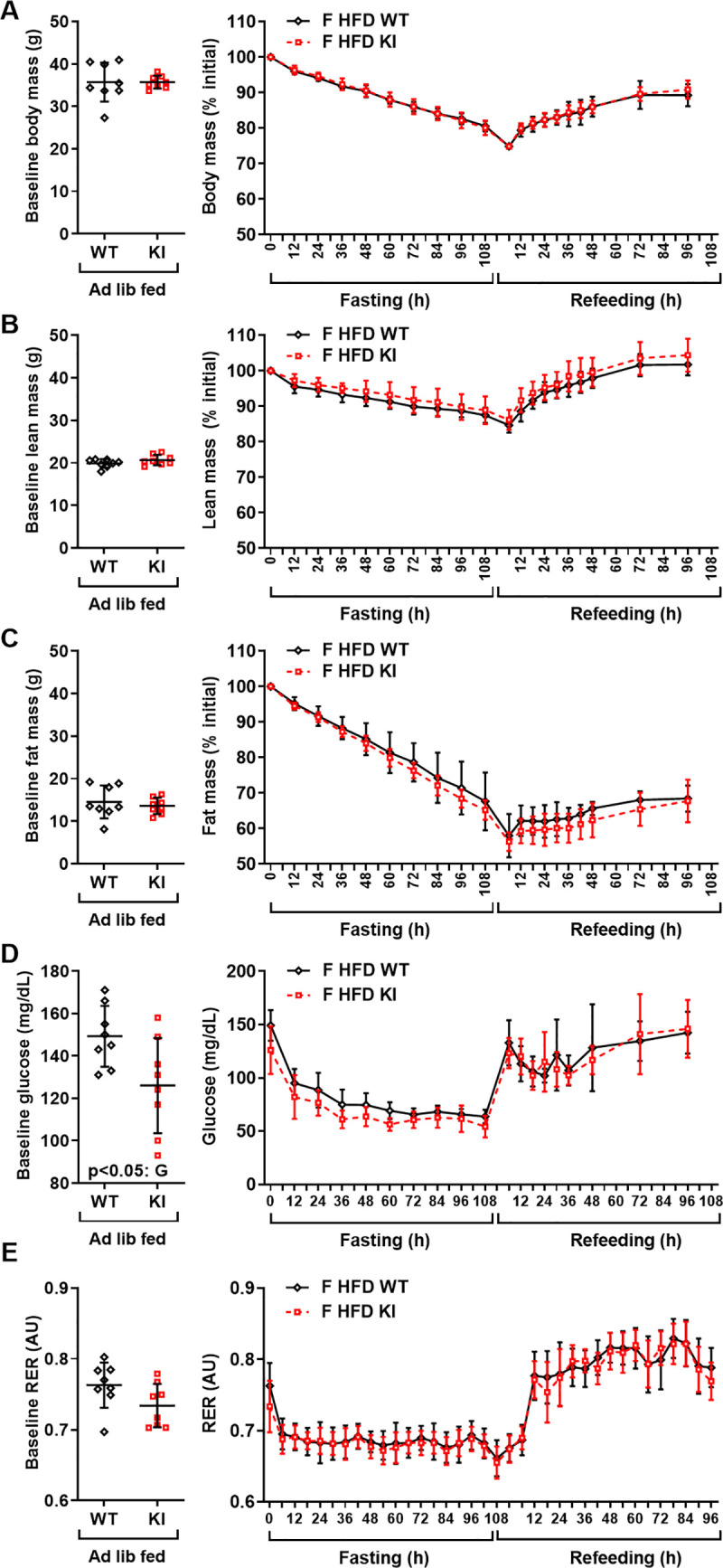
Effect of the CREBRF^R458Q^ variant energy or metabolic homeostasis in response to prolonged nutritional stress (>24h fasting) in mice. Female (F, 36-week-old, HFD-fed, n = 8/group) wild-type (WT) and knockin (KI) mice were subjected to a monitored fast until they achieved 25% loss of their initial body mass, followed by refeeding ad libitum. Body mass, lean mass, fat mass, blood glucose, and indirect calorimetry were determined during fasting and refeeding. (**A**) Baseline total body mass after ad lib feeding (**A, left**), and during prolonged fasting and refeeding (**A, right**). (**B**) Baseline lean mass after ad lib feeding (**B, left**), and during prolonged fasting and refeeding (**B, right**). (**C**) Baseline fat mass after ad lib feeding (**C, left**), and during prolonged fasting and refeeding (**C, right**). (**D**) Baseline blood glucose after ad lib feeding (**D, left**), and during prolonged fasting and refeeding (**D**, **right**). (**E**) Baseline respiratory exchange ratio (RER) after ad lib feeding (E, left), and during prolonged fasting and refeeding (**E, right**). Baseline data (**A-E, left**) were analyzed by unpaired two-tailed t-test. Indirect calorimetry data was analyzed as described in Fig 3. Other data were analyzed using general linear models with time as a within subject variable and G = genotype (WT, KI) as the fixed factor. Data are mean (SD). Main effects with a significance level of p<0.05 are indicated.

Evidence from Drosophila [18] and murine 3T3-L1 adipocytes [6] implicates CREBRF in the cellular and physiological adaptation to starvation. We, therefore, hypothesized that physiological manifestations of the murine CREBRF^R458Q^ variant might require the more extreme physiological challenge of prolonged fasting. To test this hypothesis, female WT and KI mice (F, 36-week-old, n = 8/group) were fasted until they achieved 25% weight loss, followed by refeeding ad libitum for the same duration, while closely monitoring body mass, fat mass, lean mass, blood glucose, and indirect calorimetry (**Fig 5**) [28]. At baseline, littermate weight-matched mice had comparable body mass (**Fig 5A**), lean mass (**Fig 5B**), and fat mass (**Fig 5C**). Mice responded remarkably well to prolonged starvation, exhibiting normal physical and behavioral characteristics. In response to prolonged to fasting, mice lost ~25% of their initial body mass (**Fig 5A, right**), and the rate of loss with fasting as well as rate of gain with refeeding were similar for percent total body mass (**Fig 5A**), lean mass (**Fig 5B**), and fat mass (**Fig 5C**). Thus, the CREBRF^R458Q^ variant does not differentially affect body mass or composition during prolonged fasting. We next looked at the effect of prolonged fasting on energy substrate homeostasis and utilization. In this smaller sub-cohort, there was a slightly lower baseline blood glucose (p<0.05) and RER (p = 0.09) in *Crebrf*KI mice that was inconsistent with result in the larger group (**Fig 4**), likely reflecting a cohort effect in timing since last feeding in the ad lib fed state rather than true difference between groups. Notably, during prolonged fasting, blood glucose (**Fig 5D**) and RER (**Fig 5E**) converged to similar minimums and rebounded identically after refeeding in both genotypes. Thus, the CREBRF^R458Q^ variant does not differentially affect glucose homeostasis or energy substrate utilization during prolonged fasting. Thus, overall, the CREBRF^R458Q^ variant does not affect energy or metabolic homeostasis in response to prolonged nutritional stress (>24h fasting) in mice.

### The CREBRF^R458Q^ variant does not affect energy or metabolic homeostasis in response to mTORC1 inhibition in mice

**Fig 6:**
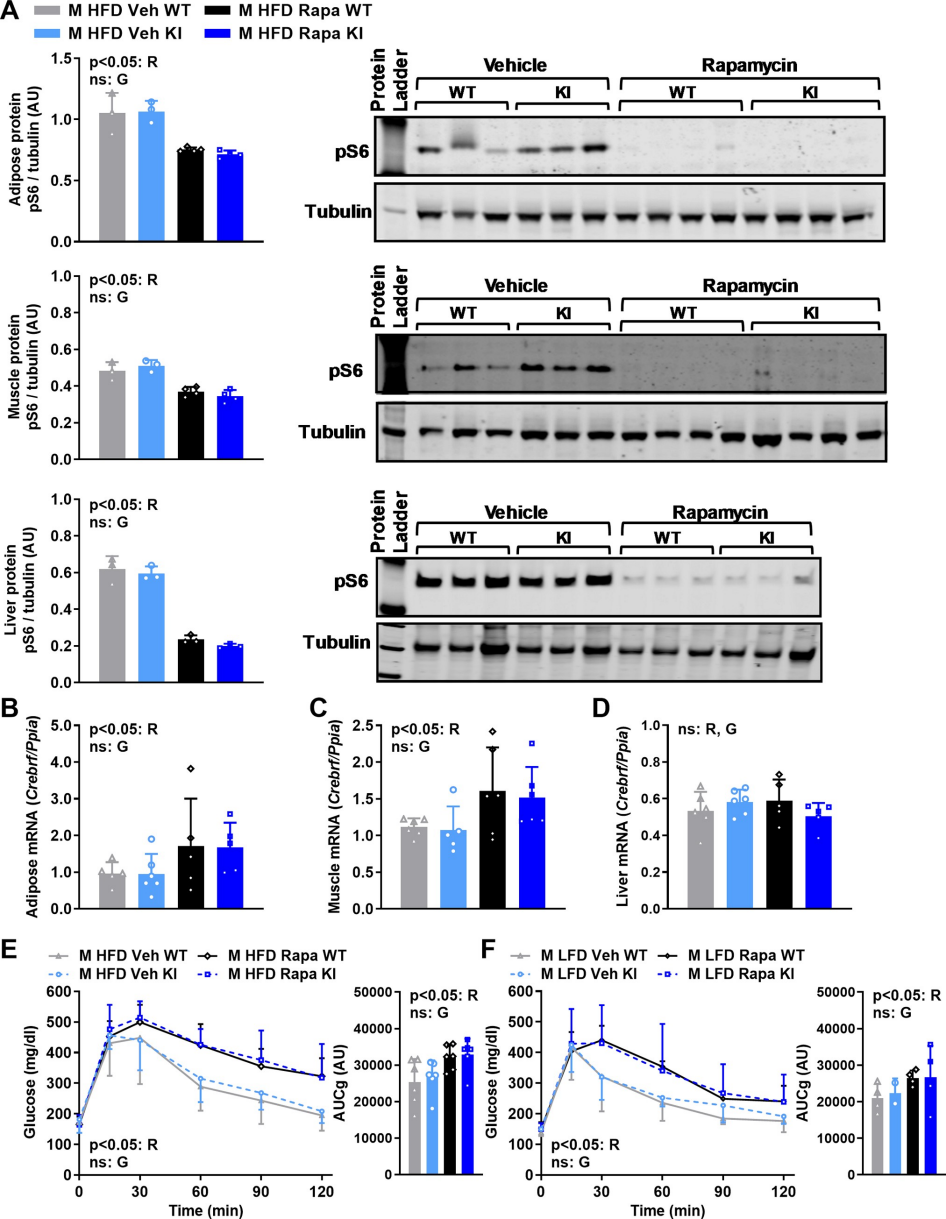
Effect of CREBRF^R458Q^ on energy or metabolic homeostasis in response to mTORC1 inhibition in mice. Male (M, n = 6/group) wild-type (WT) and knockin (KI) mice were fed low-fat diet (LFD) or high-fat diet (HFD) from weaning until sacrifice at 22 weeks of age were treated with vehicle (Veh) or rapamycin (Rapa) 4 mg/kg body weight via intraperitoneal (ip) injection every other day for three weeks prior to sacrifice at ~22 weeks of age in the ad lib fed state. **(A)** Protein expression of Ser240/244 phosphorylated ribosomal protein S6 (pS6) relative to tubulin control by Western blotting in adipose tissue (top), gastrocnemius skeletal muscle (middle), and liver (bottom) [left, quantification; right, actual blots). **(B-D)** *Crebrf* mRNA relative to *Ppia* control in adipose tissue **(B)**, gastrocnemius skeletal muscle **(C)**, and liver **(D)** of mice treated with rapamycin. **(E-F)** Glucose tolerance tests (GTTs, left) and area under the curve for glucose (AUCg, right) in rapamycin-treated HFD-fed (n = 6/group) **(E)** and LFD-fed (n = 2-4/group) **(F)** mice. General linear models with **(E-F,** GTTs**)** or without **(A-D)** time as a within subject variable were used to compare the above dependent variables between R = rapamycin treatment (rapamycin, vehicle) and G = genotype (WT, KI) as fixed factors. Data are mean (SD). Main effects with a significance level of p<0.05 are indicated. Main effects with a significance level of ≥0.05 are labeled as non-significant (ns).

The Drosophila homolog of CREBRF, REPTOR, is induced by the TORC1 inhibitor, rapamycin, and mediates the majority of the downstream transcriptional response to rapamycin-mediated TORC1 inhibition, primarily via its (de)phosphorylation-dependent translocation to the nucleus [18]. Although there is no evidence of the latter in mammalian cells, we have confirmed that rapamycin induces CREBRF mRNA expression in murine 3T3-L1 cells [6]. We, therefore, hypothesized that the physiological manifestations of the murine CREBRF^R458Q^ variant might be TORC1-specific, and therefore may be unmasked by rapamycin treatment. To test this hypothesis, we treated LFD- and HFD-fed male mice with vehicle or rapamycin 4 mg/kg body weight via intraperitoneal (ip) injection every other day for three weeks prior to sacrifice at ~22 weeks of age in the ad lib-fed state (**Fig 6**). We first confirmed the effectiveness of our pharmacological intervention. As expected, rapamycin treatment decreased phosphorylated ribosomal S6 (pS6), a known downstream target of TORC1, in all tissues examined (adipose tissue, skeletal muscle, and liver) (**Fig 6A**). We next confirmed the expression and regulation of *Crebrf* mRNA in metabolically-relevant tissues of rapamycin-treated mice (**Fig 6B–6D**). Rapamycin increased *Crebrf* mRNA expression in adipose tissue and skeletal muscle but not liver, whereas genotype did not influence *Crebrf* mRNA expression, regardless of treatment. Consistent with previous reports [22], rapamycin treatment resulted in a clear and significant impairment of glucose tolerance and increased area under the curve for glucose (AUCg) in both HFD-fed (**Fig 6E**) and LFD-fed (**Fig 6F**) mice. However, there was no effect of genotype on the glycemic response to rapamycin treatment in mice on either diet. Thus, the CREBRF^R458Q^ variant does not affect energy or metabolic homeostasis in response to mTORC1 inhibition in mice.

### The CREBRF^R458Q^ variant promotes greater loss of fat but not lean mass with age

**Fig 7:**
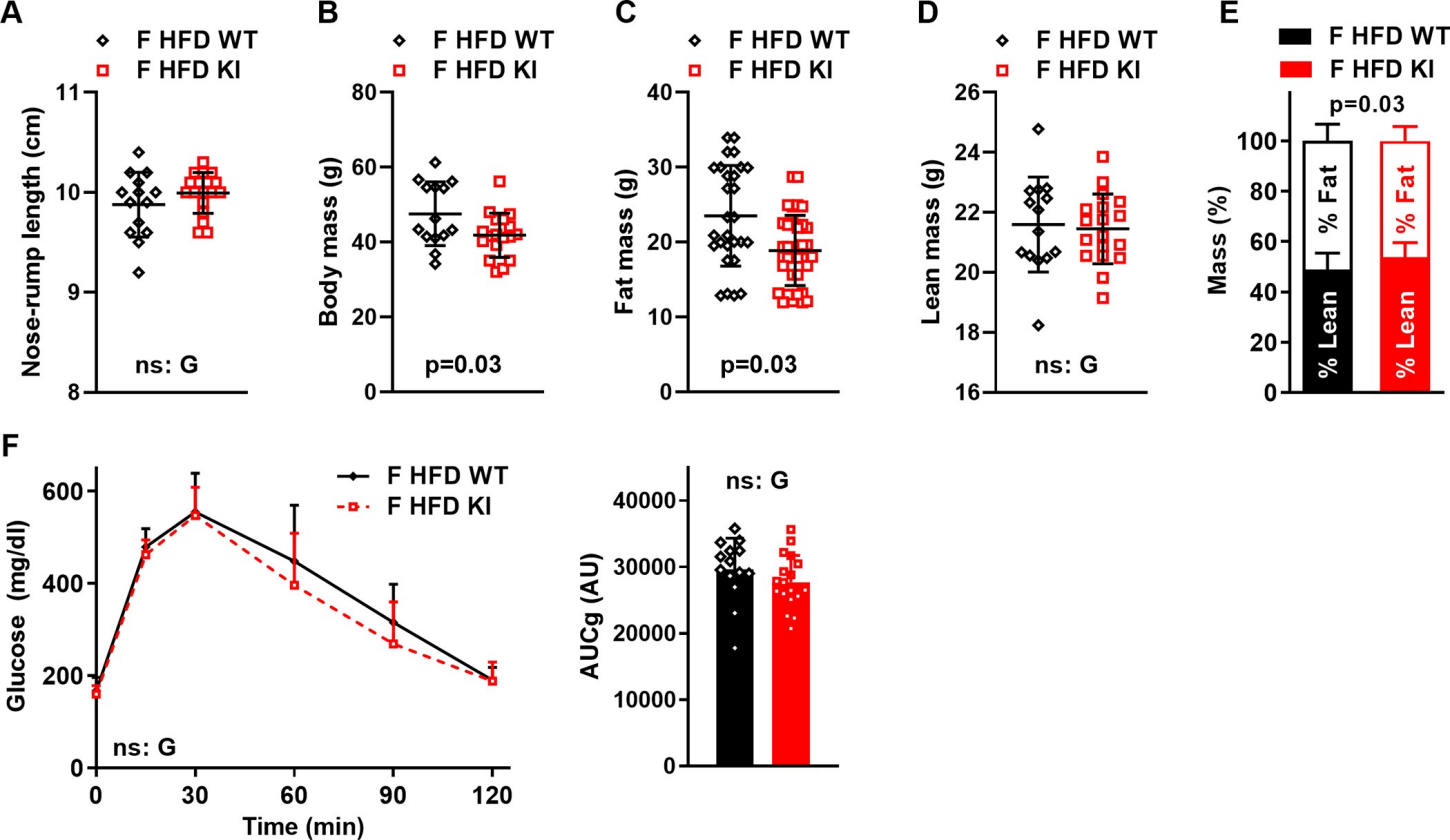
Effect of the CREBRF^R458Q^ variant energy or metabolic homeostasis in response to aging (52 weeks) in mice. Female (F, 52-week-old, HFD-fed, n = 14-18/group) wild-type (WT) and knockin (KI) mice were aged 52 weeks (i.e. 1 year). (A) Nose-rump length (cm), (B) total body mass, (C) fat mas, and (D) lean mass were determined as for younger mice. (E) Fat and lean mass expressed as percent of total body mass. (F) Glucose tolerance tests (GTTs, left) and area under the curve for glucose (AUCg, right). GTT data were analyzed by general linear models with time as a within subject variable and G = genotype (WT, KI) as the fixed factor. All other data were analyzed by unpaired two-tailed t-test. Data are mean (SD). Main effects with a significance level of ≥0.05 are labeled as non-significant (ns). Otherwise, p values are indicated.

Metabolic (and non-metabolic) phenotypes are often revealed and/or exacerbated with age. To further evaluate the effect of the CREBRF^R458Q^ variant in the context of this physiological stressor, we next followed HFD-fed female WT and KI mice to 52 weeks of age (**Fig 7**). There was no genotype effect on nose-rump length (**Fig 7A**) or lean body mass (**Fig 7D**), however, total body mass (**Fig 7B**) and fat mass (**Fig 7C**) were lower in *Crebrf*KI mice. When body composition was expressed as percent of total mass, percent lean mass was higher, and percent fat mass was lower in *Crebrf*KI mice (**Fig 7E**). There was no difference in glucose tolerance (**Fig 7F**).

## Discussion

The goal of this investigation was to determine the function and physiological relevance of the human CREBRF^R457Q^ obesity/diabetes risk variant by generating and evaluating a murine “knockin” model with the orthologous CREBRF^R458Q^ variant. However, despite strong evidence linking the CREBRF^R457Q^ variant to obesity and diabetes risk in humans, we were not able to detect any genotype effects of the CREBRF^R458Q^ variant on energy, glucose, or metabolic phenotypes in mice. Furthermore, we examined these phenotypes at baseline as well as under conditions of acute (<24h fasting/refeeding), chronic (low- and high-fat diet feeding up to 20w), and extreme (5d starvation) nutritional stress, as well as in response to pharmacological stress (inhibition of TORC1 by rapamycin) and aging (52 weeks). Of these more extreme interventions, only age revealed a small but significant decrease in fat but not lean mass in *Crebrf*KI mice. In addition to these results, we demonstrated that *Crebrf* mRNA expression is induced by fasting (adipose tissue, skeletal muscle, and liver) and rapamycin-mediated inhibition of TORC1 (adipose tissue, skeletal muscle), which has not been previously reported. The data presented suggest that the CREBRF^R458Q^ knockin mouse model is not likely to be an effective model for understanding the function and physiological relevance of this gene/variant.

There are several possible explanations for why the CREBRF^R458Q^ knockin mouse model did not recapitulate the phenotype of human CREBRF^R457Q^ carriers. We first consideration is to ensure that the model is valid. To do so, we conducted several quality control experiments to confirm that we indeed successfully engineered the desired orthologous CREBRF^R458Q^ knockin allele in mice. We confirmed this by directly sequencing the allele, as well as by developing three separate genotyping methods and by genotyping mice with at least two methods at weaning and again after sacrifice. In addition to the nonsynonymous CREBRF variant (G→A), we also introduced a BamH1 site (T→C) for genotyping purposes, however the latter is a synonymous variant that does not change the amino acids sequence of the protein and is, therefore, not expected to have any phenotypic consequences. Thus, we are confident that we evaluated a valid mouse model. As further support, a separate group recently created two separate CREBRF^R458Q^ knockin mouse models on different background strains (C57BL/6J and FVB/NJ) with remarkably similar results as our model (i.e. small or no effect on energy or glucose homeostasis) with a few exceptions: 1) a small increase in length of *Crebrf*KI FVB/NJ males that was significant at 8 weeks [10] but not 25 weeks [29], and 2) a greater loss of fat mass relative to lean mass with age (20 months) in *Crebrf*KI C57BL/6J males [29]. Although we found no difference in length in our model, our aging experiment did identify a slightly greater loss of fat mass relative to lean mass in *Crebrf*KI mice, albeit under different conditions (F, HFD, 52 weeks). Neither aging study was comprehensive, and effect sizes were small. Additional interventions (i.e. more extreme aging, exercise, cachexia) could be conducted to more rigorously tests the hypothesis that the CREBRF^R458Q^ variant preferentially enhances/preserves lean relative to fat mass. In addition to experimental conditions, it is possible that small effect sizes and/or compensatory mechanisms may have contributed to the inability to detect a robust phenotype in CREBRF^R458Q^ knockin mice. A strength of our study is that we rigorously tested a variety of physiological as well as more extreme conditions. Even without a robust metabolic phenotype in mice, it remains possible that the CREBRF^R457Q^ variant is causal/relevant in humans.

Alternatively, it is also possible that the human CREBRF^R457Q^ variant is not the causal variant contributing to obesity and diabetes risk in humans. We do not believe this to be the case for two reasons: 1) the human CREBRF^R457Q^ variant is strongly and repeatedly linked to metabolic traits in humans (especially BMI, glucose, and height), and 2) CREBRF is linked to multiple biological processes known to contribute to these metabolic traits. Regarding the former, in our original report, we confirmed the relationship between the CREBRF^R457Q^ variant and BMI in an initial cohort of 3,072 Samoans and in a separate replication cohort of 2,102 additional Samoans. This relationship between the CREBRF^R457Q^ variant and BMI was highly significant (β 1.356, p 1.12 x 10^−13^), as were the associations with obesity risk (OR 1.305, p 1.12 x 10^−5^) and diabetes risk (OR 0.586, p 6.68 x 10^−9^ after adjusting for BMI) [6]. The relationship between CREBRF^R457Q^ and obesity/diabetes risk has now been replicated by numerous groups in other populations across Oceania [9, 11–15]. These data provide strong support for the observed associations, but are not sufficient to prove causality.

It is possible that one or more variants in linkage disequilibrium with the CREBRF^R457Q^ variant, rather than (or in combination with) the CREBRF^R457Q^ variant itself may be the primary driver(s) of obesity/diabetes risk in humans. In our original report, we actually identified several variants in proximity to CREBRF that were in high linkage disequilibrium [6]. We concentrated on three variants: 1) rs12513649—located 35,727 bp upstream of the CREBRF start site (between the ATP6V0E1 and CREBRF genes), 2) rs150207780 –located in the intronic region between exon1 and exon 2 of CREBRF, 3) rs373863828 (i.e., CREBRF^R457Q^)–located in a highly conserved region (GERP score 5.49) in exon 5 of CREBRF. Since CREBRF^R457Q^ was a missense variant that was predicted to be highly damaging (SIFT, 0.03; PolyPhen-2, 0.996), it was the leading candidate. In addition, a potential causal role is supported by the aforementioned data in murine 3T3-L1 adipocytes. In considering the other variants noted above, both rs12513649 and rs150207780 are both present at minor allele frequencies of 6.8% and 7.7% in East Asian individuals without rs373863828 (5-173045049-C-G, 5-173079668-C-T and 5-173108771-G-A, respectively, in gnomAD 3.1 [30], but no association between them and BMI [31, 32] or T2D [33] has been reported. Though variants far upstream and/or in introns are less likely to contribute to phenotypes, additional studies of these and other variants in linkage disequilibrium with the CREBRF^R457Q^ variant, alone and in combination, may be required to clarify their individual and combined effects in human and mouse cells.

Regarding the biological evidence linking CREBRF and/or the CREBRF^R457Q^ variant to metabolism, we have previously demonstrated that ectopic overexpression of human CREBRF^R457Q^ in a murine 3T3-L1 adipocyte model promotes adipogenesis, decreases mitochondrial respiration, and increases fat storage [6], consistent with the observed whole-body phenotypes in human carriers of the CREBRF^R457Q^ allele, and providing functional support for a causal role of this variant. Although additional mechanistic studies of CREBRF^R457Q^ variant are still underway, multiple studies have emerged linking (wild-type) CREBRF to multiple proteins/pathways known to be critically involved in energy and metabolic homeostasis including CREB-family proteins [34, 35], TORC1 [18, 36], Akt [37], and glucocorticoid receptor [17, 38] across different species (i.e., human, mouse, goat, Drosophila) and cell types (i.e., adipocyte, neurons, gastric cells, endometrial cells, etc.). In vivo, genetic manipulation of endogenous CREBRF in mice and Drosophila results in dramatic phenotypes related to energy and metabolic homeostasis [17, 18]. Specifically, global deletion of CREBRF in mice or Drosophila decreases body mass, whereas the opposite phenotype is observed in human carriers of the CREBRF^R457Q^ allele. We have separately confirmed that global deletion of murine CREBRF in mice decreases body mass and protects against diet-induced obesity, whereas global overexpression of murine CREBRF in mice has the opposite effect (Kershaw, publication in progress). These data suggest that CREBRF does indeed contribute to energy and metabolic homeostasis, and that global and/or tissue-specific targeted deletion and/or overexpression of endogenous murine CREBRF may be more useful mouse models to understand the complexities of this gene. Such studies could lead to more targeted mechanistic hypotheses related to the CREBRF^R457Q^ variant that could be tested using cellular models, especially “knockin” (rather than ectopic expression) models of CREBRF^R457Q^ in human cell lines.

Despite these findings, CREBRF remains a potentially important yet poorly understood gene/protein linked to human health and disease. Thus far, the CREBRF^R457Q^ variant is associated with increased obesity risk and decreased diabetes risk in humans [6, 9, 11–14] as well as height [8, 10] and body composition/lean mass [39]. However, the endogenous wild-type (non-variant) CREBRF gene/protein has been linked, either directly or indirectly, to many other clinically-relevant physiological and/or pathological processes in mammals, including susceptibility to viral infection [34], endometrial function during pregnancy [36], angiogenesis [40], neuroendocrine function and behavior [17], Alzheimer’s disease [41], and cancer [37, 42–46]. Thus, there continues to be an urgent need to understand the mechanism by which this gene/protein contributes to normal biology and disease, and in particular, the specific effect of the CREBRF^R457Q^ variant, which is highly prevalent in Oceanic populations.

In summary, the CREBRF^R458Q^ knockin mouse model of the human CREBRF^R457Q^ obesity-risk variant does not influence energy or glucose homeostasis in response to nutritional stress. To better understand the physiological relevance of CREBRF, future studies could focus on murine models with global or tissues-specific deletion or overexpression of endogenous CREBRF. Such studies are likely to reveal novel insights into “normal” CREBRF function that would permit more targeted hypothesis testing related to the CREBRF variant in the future. In addition, studies examining the human CREBRF^R457Q^ variant (or variants) in human cells will be instrumental in confirming causality and well as dissecting the underlying mechanisms. Finally, additional studies in human carriers of the CREBRF^R457Q^ variant would enhance the deeper and broader understanding of how this variant might impact human health in the quest to identify tangible strategies for prevention and treatment of obesity and associated metabolic abnormalities in humans.

## Supporting information

S1 Table Oligonucleotide information. (XLSX)

S2 Table Antibody information. (XLSX)

S3 Table Key reagent information. (XLSX)

S1 Fig Comparison of human and mouse CREBRF protein sequence. **(A)** Illustration of the amino acid change from an arginine (Arg, R) to a glutamine (Gln, Q) at position 457 in humans (Rs373863828) or 458 in mouse. **(B)** The human and mouse protein sequences are on the top and bottom line, respectively, with the comparison between the two sequences in the middle. Overall, the human and mouse CREBRF protein sequence are highly homologous (~94%). The human R457 corresponds to mouse R458 (shown in RED).
(TIF)

S2 Fig Effect of the CREBRF^R458Q^ variant on tissue weights at the time of sacrifice in response to nutritional and pharmacological interventions. **(A-B)** Tissue weights following acute and chronic nutritional interventions. Male **(A)** and female **(B)** wildtype (WT) and Crebrf^R458Q^ knockin (KI) mice were fed low-fat diet (LFD) or high-fat diet (LFD) from weaning until 22 weeks of age and then sacrificed in the fasted state (16h fast) or refed state (24h fast followed by 16h refeeding) (M, n = 7–9 per group; F 7–11 per group). General linear models were used to compare each tissue (dependent variables) between D = diet (LFD, HFD), N = nutritional status (fasted, refed), and G = genotype (WT, KI) as fixed factors. Data are mean (SD). Main effects with a significance level of p<0.05 are indicated.
(TIF)

S3 Fig Original gels. (PDF)

S1 File Dataset. (XLSX)

## References

[1] Locke AE, Kahali B, Berndt SI, Justice AE, Pers TH, Day FR, et al. Genetic studies of body mass index yield new insights for obesity biology. Nature. 2015;518(7538):197–206. doi: 10.1038/nature14177 ; PubMed Central PMCID: PMC4382211. 25673413 PMC4382211

[2] Winkler TW, Justice AE, Graff M, Barata L, Feitosa MF, Chu S, et al. The Influence of Age and Sex on Genetic Associations with Adult Body Size and Shape: A Large-Scale Genome-Wide Interaction Study. PLoS genetics. 2015;11(10):e1005378. Epub 2015/10/02. doi: 10.1371/journal.pgen.1005378; PubMed Central PMCID: PMC4591371. 26426971 PMC4591371

[3] Shungin D, Winkler TW, Croteau-Chonka DC, Ferreira T, Locke AE, Magi R, et al. New genetic loci link adipose and insulin biology to body fat distribution. Nature. 2015;518(7538):187–96. doi: 10.1038/nature14132 ; PubMed Central PMCID: PMC4338562. 25673412 PMC4338562

[4] Fuchsberger C, Flannick J, Teslovich TM, Mahajan A, Agarwala V, Gaulton KJ, et al. The genetic architecture of type 2 diabetes. Nature. 2016;536(7614):41–7. Epub 2016/07/12. doi: 10.1038/nature18642 ; PubMed Central PMCID: PMC5034897. 27398621 PMC5034897

[5] Mills MC, Rahal C. The GWAS Diversity Monitor tracks diversity by disease in real time. Nature genetics. 2020;52(3):242–3. Epub 2020/03/07. doi: 10.1038/s41588-020-0580-y . 32139905

[6] Minster RL, Hawley NL, Su CT, Sun G, Kershaw EE, Cheng H, et al. A thrifty variant in CREBRF strongly influences body mass index in Samoans. Nature genetics. 2016;48(9):1049–54. Epub 2016/07/28. doi: 10.1038/ng.3620 ; PubMed Central PMCID: PMC5069069. 27455349 PMC5069069

[7] Loos RJ. CREBRF variant increases obesity risk and protects against diabetes in Samoans. Nature genetics. 2016;48(9):976–8. doi: 10.1038/ng.3653 . 27573685

[8] Carlson JC, Rosenthal SL, Russell EM, Hawley NL, Sun G, Cheng H, et al. A missense variant in CREBRF is associated with taller stature in Samoans. Am J Hum Biol. 2020:e23414. Epub 2020/03/20. doi: 10.1002/ajhb.23414. 32190945 PMC7501196

[9] Berry SD, Walker CG, Ly K, Snell RG, Atatoa Carr PE, Bandara D, et al. Widespread prevalence of a CREBRF variant amongst Maori and Pacific children is associated with weight and height in early childhood. International journal of obesity. 2018;42(4):603–7. Epub 2017/09/21. doi: 10.1038/ijo.2017.230 . 28928463

[10] Metcalfe LK, Krishnan M, Turner N, Yaghootkar H, Merry TL, Dewes O, et al. The Maori and Pacific specific CREBRF variant and adult height. International journal of obesity. 2020;44(3):748–52. Epub 2019/09/24. doi: 10.1038/s41366-019-0437-6 . 31543511

[11] Naka I, Furusawa T, Kimura R, Natsuhara K, Yamauchi T, Nakazawa M, et al. A missense variant, rs373863828-A (p.Arg457Gln), of CREBRF and body mass index in Oceanic populations. Journal of human genetics. 2017;62(9):847–9. Epub 2017/04/14. doi: 10.1038/jhg.2017.44 . 28405013

[12] Krishnan M, Major TJ, Topless RK, Dewes O, Yu L, Thompson JMD, et al. Discordant association of the CREBRF rs373863828 A allele with increased BMI and protection from type 2 diabetes in Maori and Pacific (Polynesian) people living in Aotearoa/New Zealand. Diabetologia. 2018;61(7):1603–13. Epub 2018/05/04. doi: 10.1007/s00125-018-4623-1 ; PubMed Central PMCID: PMC6434933. 29721634 PMC6434933

[13] Hanson RL, Safabakhsh S, Curtis JM, Hsueh WC, Jones LI, Aflague TF, et al. Association of CREBRF variants with obesity and diabetes in Pacific Islanders from Guam and Saipan. Diabetologia. 2019;62(9):1647–52. Epub 2019/07/08. doi: 10.1007/s00125-019-4932-z ; PubMed Central PMCID: PMC6721609. 31280340 PMC6721609

[14] Krishnan M, Murphy R, Okesene-Gafa KAM, Ji M, Thompson JMD, Taylor RS, et al. The Pacific-specific CREBRF rs373863828 allele protects against gestational diabetes mellitus in Maori and Pacific women with obesity. Diabetologia. 2020;63(10):2169–76. Epub 2020/07/13. doi: 10.1007/s00125-020-05202-8 . 32654027

[15] Lin M, Caberto C, Wan P, Li Y, Lum-Jones A, Tiirikainen M, et al. Population-specific reference panels are crucial for genetic analyses: an example of the CREBRF locus in Native Hawaiians. Human molecular genetics. 2020;29(13):2275–84. Epub 2020/06/04. doi: 10.1093/hmg/ddaa083 ; PubMed Central PMCID: PMC7399533. 32491157 PMC7399533

[16] Audas TE, Li Y, Liang G, Lu R. A novel protein, Luman/CREB3 recruitment factor, inhibits Luman activation of the unfolded protein response. Molecular and cellular biology. 2008;28(12):3952–66. doi: 10.1128/MCB.01439-07 ; PubMed Central PMCID: PMC2423117. 18391022 PMC2423117

[17] Martyn AC, Choleris E, Gillis DJ, Armstrong JN, Amor TR, McCluggage AR, et al. Luman/CREB3 recruitment factor regulates glucocorticoid receptor activity and is essential for prolactin-mediated maternal instinct. Molecular and cellular biology. 2012;32(24):5140–50. Epub 2012/10/17. doi: 10.1128/MCB.01142-12 ; PubMed Central PMCID: PMC3510545. 23071095 PMC3510545

[18] Tiebe M, Lutz M, De La Garza A, Buechling T, Boutros M, Teleman AA. REPTOR and REPTOR-BP Regulate Organismal Metabolism and Transcription Downstream of TORC1. Developmental cell. 2015;33(3):272–84. doi: 10.1016/j.devcel.2015.03.013 . 25920570 PMC4430829

[19] Pelletier S, Gingras S, Green DR. Mouse genome engineering via CRISPR-Cas9 for study of immune function. Immunity. 2015;42(1):18–27. Epub 2015/01/22. doi: 10.1016/j.immuni.2015.01.004 ; PubMed Central PMCID: PMC4720985. 25607456 PMC4720985

[20] Wang H, Yang H, Shivalila CS, Dawlaty MM, Cheng AW, Zhang F, et al. One-step generation of mice carrying mutations in multiple genes by CRISPR/Cas-mediated genome engineering. Cell. 2013;153(4):910–8. Epub 2013/05/07. doi: 10.1016/j.cell.2013.04.025 ; PubMed Central PMCID: PMC3969854. 23643243 PMC3969854

[21] Falabella M, Sun L, Barr J, Pena AZ, Kershaw EE, Gingras S, et al. Single-Step qPCR and dPCR Detection of Diverse CRISPR-Cas9 Gene Editing Events In Vivo. G3 (Bethesda). 2017;7(10):3533–42. Epub 2017/09/02. doi: 10.1534/g3.117.300123 ; PubMed Central PMCID: PMC5633400. 28860183 PMC5633400

[22] Fang Y, Westbrook R, Hill C, Boparai RK, Arum O, Spong A, et al. Duration of rapamycin treatment has differential effects on metabolism in mice. Cell metabolism. 2013;17(3):456–62. Epub 2013/03/12. doi: 10.1016/j.cmet.2013.02.008 ; PubMed Central PMCID: PMC3658445. 23473038 PMC3658445

[23] Basantani MK, Sitnick MT, Cai L, Brenner DS, Gardner NP, Li JZ, et al. Pnpla3/Adiponutrin deficiency in mice does not contribute to fatty liver disease or metabolic syndrome. Journal of lipid research. 2011;52(2):318–29. doi: 10.1194/jlr.M011205 ; PubMed Central PMCID: PMC3023552. 21068004 PMC3023552

[24] Sitnick MT, Basantani MK, Cai L, Schoiswohl G, Yazbeck CF, Distefano G, et al. Skeletal muscle triacylglycerol hydrolysis does not influence metabolic complications of obesity. Diabetes. 2013;62(10):3350–61. doi: 10.2337/db13-0500 ; PubMed Central PMCID: PMC3781480. 23835334 PMC3781480

[25] Schoiswohl G, Stefanovic-Racic M, Menke MN, Wills RC, Surlow BA, Basantani MK, et al. Impact of reduced ATGL-mediated adipocyte lipolysis on obesity-associated insulin resistance and inflammation in male mice. Endocrinology. 2015;In press. doi: 10.1210/en.2015-1322 26196542 PMC4588821

[26] Bustin SA, Benes V, Garson JA, Hellemans J, Huggett J, Kubista M, et al. The MIQE guidelines: minimum information for publication of quantitative real-time PCR experiments. Clin Chem. 2009;55(4):611–22. Epub 2009/02/28. clinchem.2008.112797 [pii] doi: 10.1373/clinchem.2008.112797 . 19246619

[27] Kienesberger PC, Lee D, Pulinilkunnil T, Brenner DS, Cai L, Magnes C, et al. Adipose triglyceride lipase deficiency causes tissue-specific changes in insulin signaling. The Journal of biological chemistry. 2009;284(44):30218–29. doi: 10.1074/jbc.M109.047787 ; PubMed Central PMCID: PMC2781577. 19723629 PMC2781577

[28] Jensen TL, Kiersgaard MK, Sorensen DB, Mikkelsen LF. Fasting of mice: a review. Lab Anim. 2013;47(4):225–40. Epub 2013/09/13. doi: 10.1177/0023677213501659 . 24025567

[29] Lee K, Vakili S, Burden HJ, Adams S, Smith GC, Kulatea B, et al. The minor allele of the CREBRF rs373863828 p.R457Q coding variant is associated with reduced levels of myostatin in males: Implications for body composition. medRxiv. 2021:2021.07.13.21260462. doi: 10.1101/2021.07.13.21260462 PMC8927835 35218947

[30] Karczewski KJ, Francioli LC, Tiao G, Cummings BB, Alfoldi J, Wang Q, et al. The mutational constraint spectrum quantified from variation in 141,456 humans. Nature. 2020;581(7809):434–43. Epub 2020/05/29. doi: 10.1038/s41586-020-2308-7 ; PubMed Central PMCID: PMC7334197. 32461654 PMC7334197

[31] Wen W, Zheng W, Okada Y, Takeuchi F, Tabara Y, Hwang JY, et al. Meta-analysis of genome-wide association studies in East Asian-ancestry populations identifies four new loci for body mass index. Human molecular genetics. 2014;23(20):5492–504. Epub 2014/05/28. doi: 10.1093/hmg/ddu248 ; PubMed Central PMCID: PMC4168820. 24861553 PMC4168820

[32] Cho YS, Go MJ, Kim YJ, Heo JY, Oh JH, Ban HJ, et al. A large-scale genome-wide association study of Asian populations uncovers genetic factors influencing eight quantitative traits. Nature genetics. 2009;41(5):527–34. Epub 2009/04/28. doi: 10.1038/ng.357 . 19396169

[33] Spracklen CN, Horikoshi M, Kim YJ, Lin K, Bragg F, Moon S, et al. Identification of type 2 diabetes loci in 433,540 East Asian individuals. Nature. 2020;582(7811):240–5. Epub 2020/06/06. doi: 10.1038/s41586-020-2263-3 ; PubMed Central PMCID: PMC7292783. 32499647 PMC7292783

[34] Audas TE, Hardy-Smith PW, Penney J, Taylor T, Lu R. Characterization of nuclear foci-targeting of Luman/CREB3 recruitment factor (LRF/CREBRF) and its potential role in inhibition of herpes simplex virus-1 replication. European journal of cell biology. 2016;95(12):611–22. doi: 10.1016/j.ejcb.2016.10.006 . 28029379

[35] Tiebe M, Lutz M, Senyilmaz Tiebe D, Teleman AA. Crebl2 regulates cell metabolism in muscle and liver cells. Scientific reports. 2019;9(1):19869. Epub 2019/12/29. doi: 10.1038/s41598-019-56407-w; PubMed Central PMCID: PMC6934747. 31882710 PMC6934747

[36] Yang D, Jiang T, Liu J, Zhang B, Lin P, Chen H, et al. CREB3 Regulatory Factor -mTOR-autophagy regulates goat endometrial function during early pregnancy. Biology of reproduction. 2018. Epub 2018/02/16. doi: 10.1093/biolre/ioy044. 29447354

[37] Han J, Zhang L, Zhang J, Jiang Q, Tong D, Wang X, et al. CREBRF promotes the proliferation of human gastric cancer cells via the AKT signaling pathway. Cellular and molecular biology. 2018;64(5):40–5. Epub 2018/05/08. . 29729692

[38] Penney J, Taylor T, MacLusky N, Lu R. LUMAN/CREB3 Plays a Dual Role in Stress Responses as a Cofactor of the Glucocorticoid Receptor and a Regulator of Secretion. Front Mol Neurosci. 2018;11:352. Epub 2018/10/20. doi: 10.3389/fnmol.2018.00352; PubMed Central PMCID: PMC6179040. 30337854 PMC6179040

[39] Arslanian KJ, Fidow UT, Atanoa T, Unasa-Apelu F, Naseri T, Wetzel AI, et al. A missense variant in CREBRF, rs373863828, is associated with fat-free mass, not fat mass in Samoan infants. International journal of obesity. 2021;45(1):45–55. Epub 2020/09/05. doi: 10.1038/s41366-020-00659-4 . 32884101 PMC8329753

[40] Wong NKP, Cheung H, Solly EL, Vanags LZ, Ritchie W, Nicholls SJ, et al. Exploring the Roles of CREBRF and TRIM2 in the Regulation of Angiogenesis by High-Density Lipoproteins. International journal of molecular sciences. 2018;19(7). Epub 2018/07/01. doi: 10.3390/ijms19071903; PubMed Central PMCID: PMC6073236. 29958463 PMC6073236

[41] Soleimani Zakeri NS, Pashazadeh S, MotieGhader H. Gene biomarker discovery at different stages of Alzheimer using gene co-expression network approach. Scientific reports. 2020;10(1):12210. Epub 2020/07/24. doi: 10.1038/s41598-020-69249-8; PubMed Central PMCID: PMC7376049. 32699331 PMC7376049

[42] Xue H, Zhang J, Guo X, Wang J, Li J, Gao X, et al. CREBRF is a potent tumor suppressor of glioblastoma by blocking hypoxia-induced autophagy via the CREB3/ATG5 pathway. International journal of oncology. 2016;49(2):519–28. doi: 10.3892/ijo.2016.3576 . 27278737

[43] Xue H, Yuan G, Guo X, Liu Q, Zhang J, Gao X, et al. A novel tumor-promoting mechanism of IL6 and the therapeutic efficacy of tocilizumab: Hypoxia-induced IL6 is a potent autophagy initiator in glioblastoma via the p-STAT3-MIR155-3p-CREBRF pathway. Autophagy. 2016;12(7):1129–52. doi: 10.1080/15548627.2016.1178446 ; PubMed Central PMCID: PMC4990999. 27163161 PMC4990999

[44] Wu K, Huang J, Xu T, Ye Z, Jin F, Li N, et al. MicroRNA-181b blocks gensenoside Rg3-mediated tumor suppression of gallbladder carcinoma by promoting autophagy flux via CREBRF/CREB3 pathway. Am J Transl Res. 2019;11(9):5776–87. Epub 2019/10/22. ; PubMed Central PMCID: PMC6789245. 31632547 PMC6789245

[45] Han F, Zhong C, Li W, Wang R, Zhang C, Yang X, et al. hsa_circ_0001947 suppresses acute myeloid leukemia progression via targeting hsa-miR-329-5p/CREBRF axis. Epigenomics. 2020;12(11):935–53. Epub 2020/07/14. doi: 10.2217/epi-2019-0352 . 32657138

[46] Feng S, Liu N, Chen X, Liu Y, An J. Long non-coding RNA NEAT1/miR-338-3p axis impedes the progression of acute myeloid leukemia via regulating CREBRF. Cancer Cell Int. 2020;20:112. Epub 2020/04/14. doi: 10.1186/s12935-020-01182-2; PubMed Central PMCID: PMC7137299. 32280304 PMC7137299

