# Supplementary figures and images for: A murine model of the human CREBRFR457Q obesity-risk variant does not influence energy or glucose homeostasis in response to nutritional stress
Source: PLoS One. 2021 Sep 14;16(9):e0251895. doi: 10.1371/journal.pone.0251895 (PMC8439463; doi:10.1371/journal.pone.0251895)

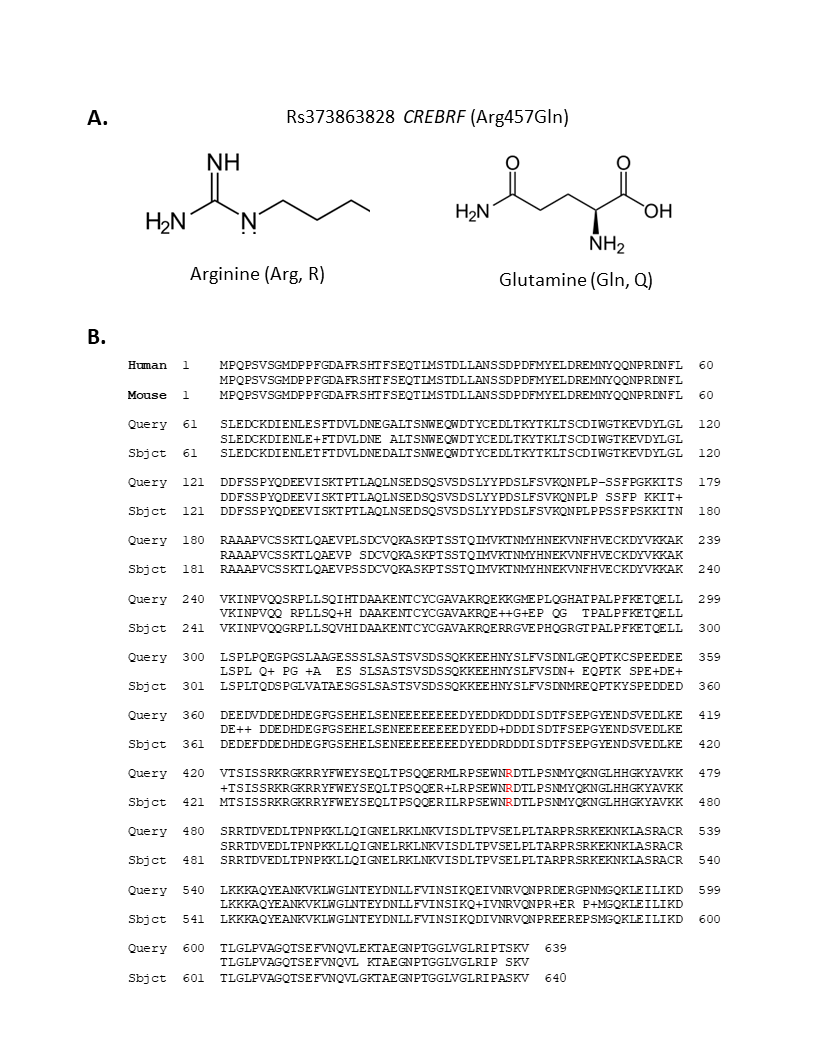

Supplement: S1 Fig — (A) Illustration of the amino acid change from an arginine (Arg, R) to a glutamine (Gln, Q) at position 457 in humans (Rs373863828) or 458 in mouse. (B) The human and mouse protein sequences are on the top and bottom line, respectively, with the comparison between the two sequences in the middle. Overall, the human and mouse CREBRF protein sequence are highly homologous (~94%). The human R457 corresponds to mouse R458 (shown in RED). (TIF) [file pone.0251895.s004.tif]
