# Supplementary material for: A murine model of the human CREBRFR457Q obesity-risk variant does not influence energy or glucose homeostasis in response to nutritional stress
Source: PLoS One. 2021 Sep 14;16(9):e0251895. doi: 10.1371/journal.pone.0251895 (PMC8439463; doi:10.1371/journal.pone.0251895)

Original / uncropped PCR gel
Fig 1B

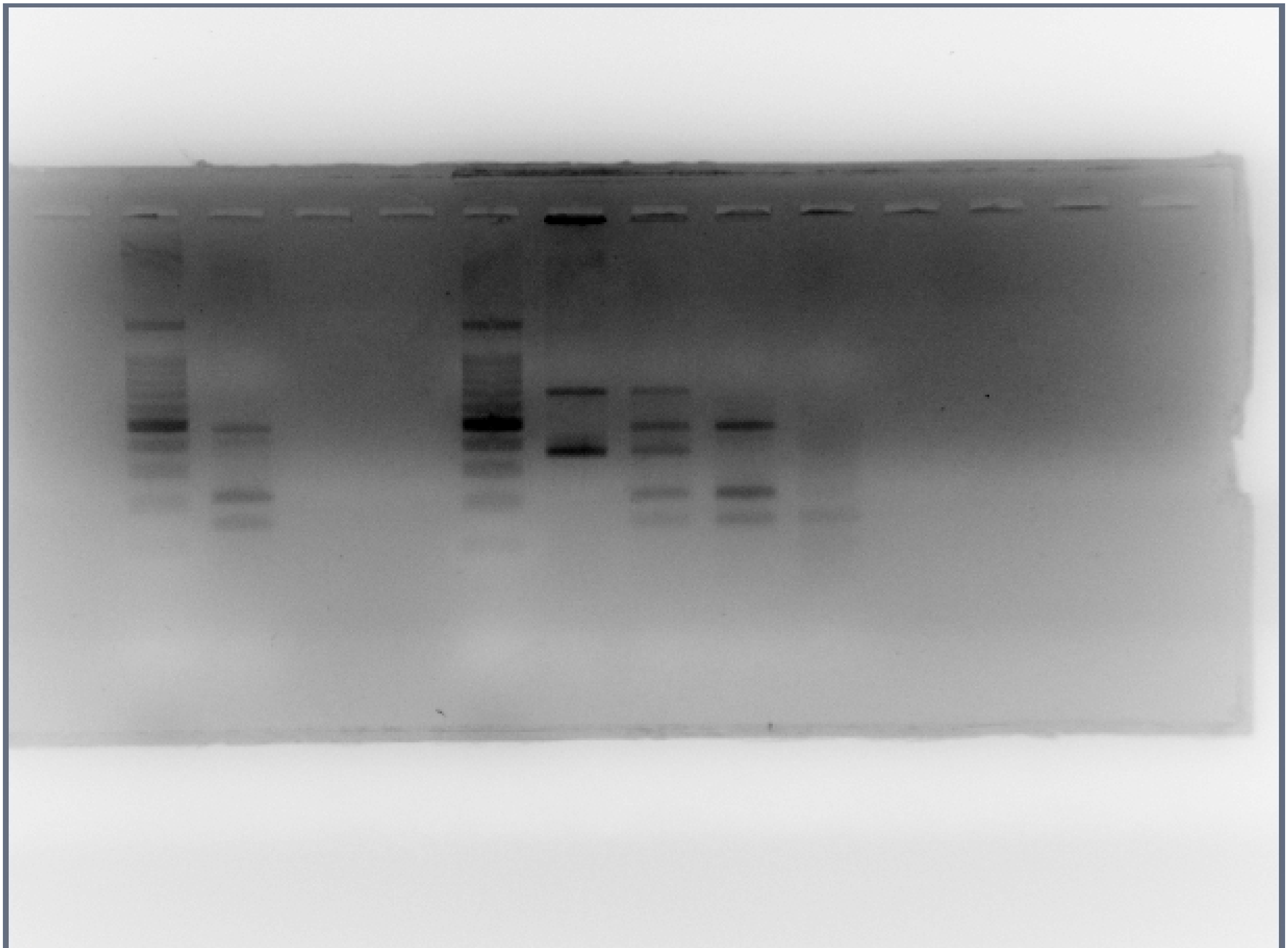

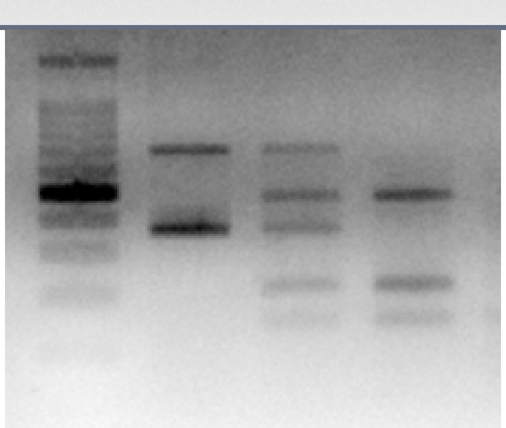

Original / uncropped Western blots
Fig 6A Adipose

Top of gel

Tubulin
~ 52kD

pS6
~ 32kD

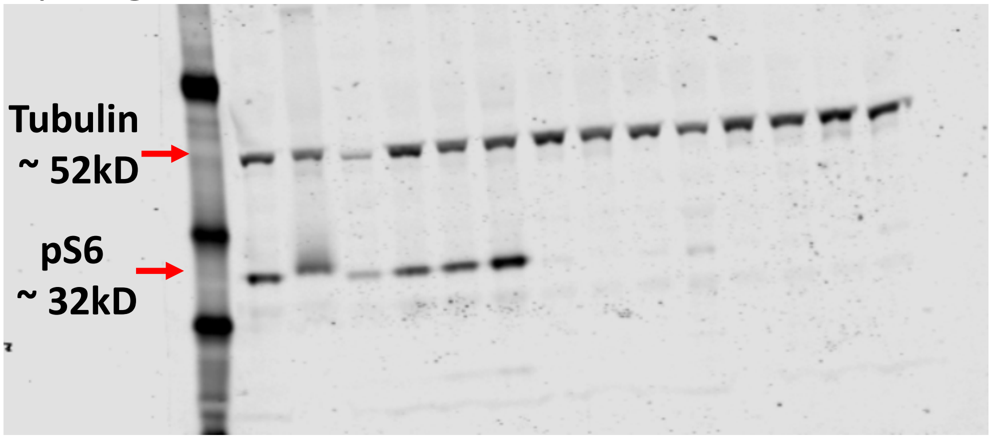

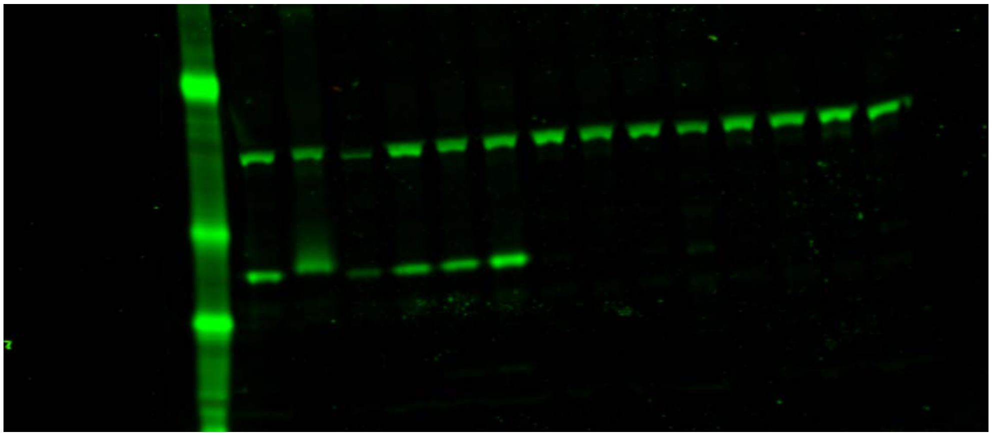

pS6
~ 32kD

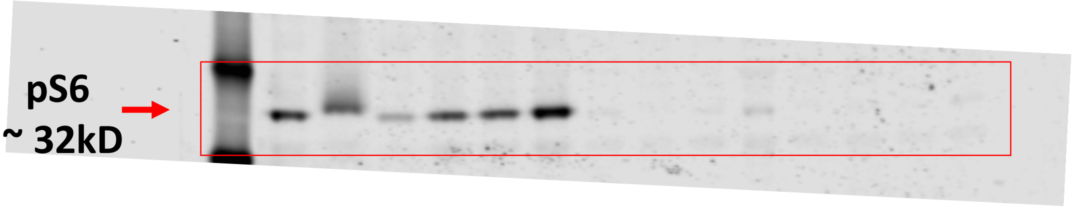

Tubulin
~ 52kD

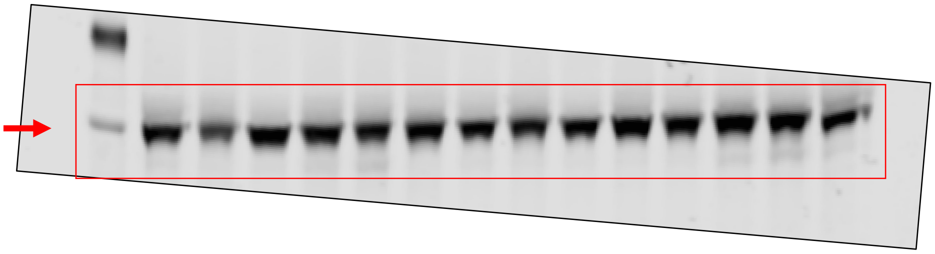

Original / uncropped Western blots
Fig 6A Muscle

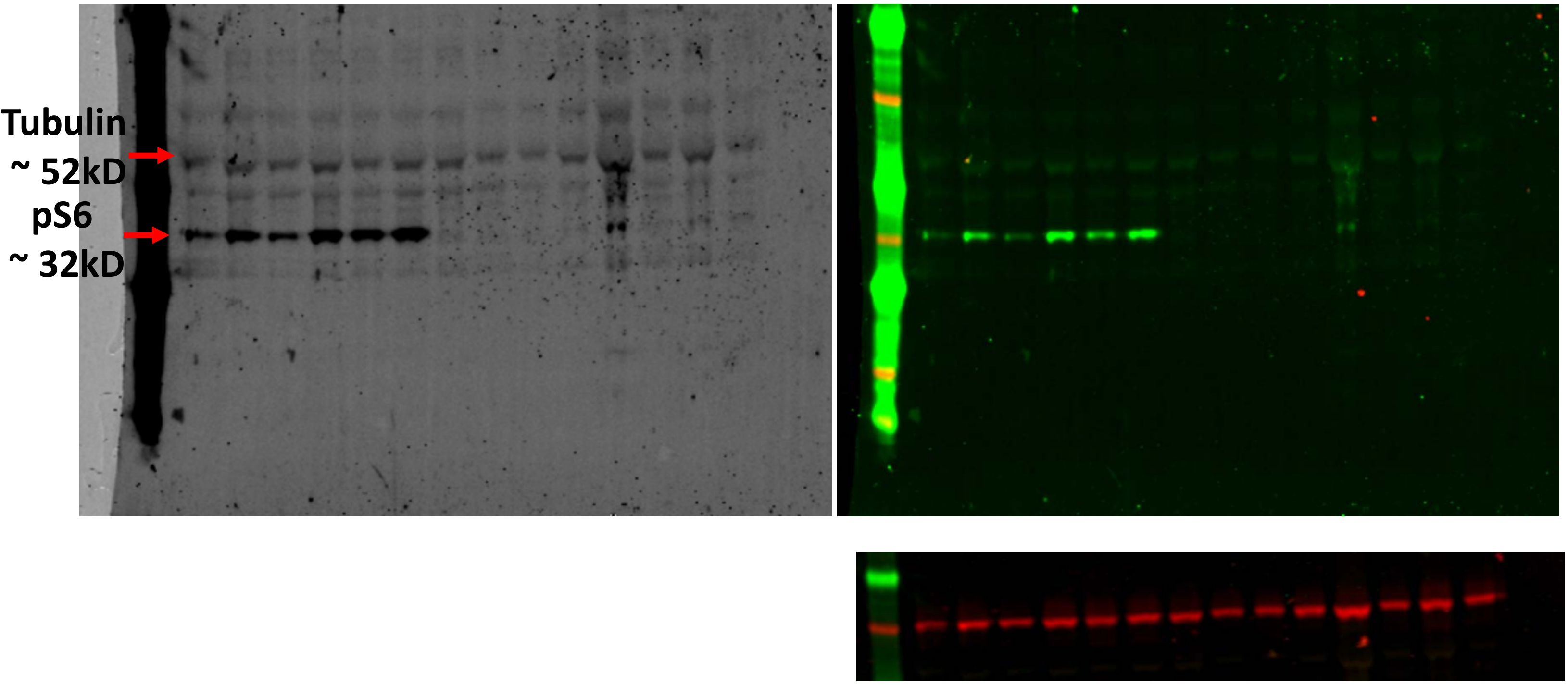

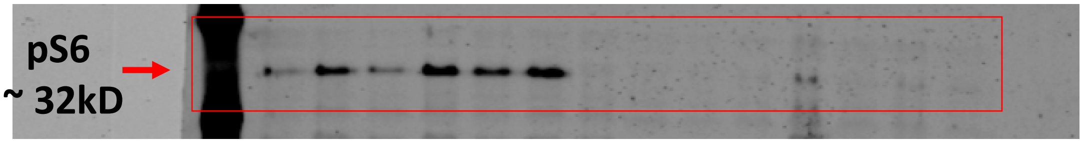

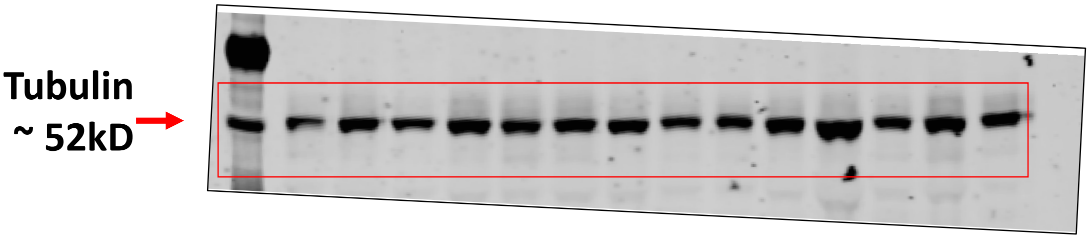

Original / uncropped Western blots
Fig 6A Liver

pS6
~ 32kD

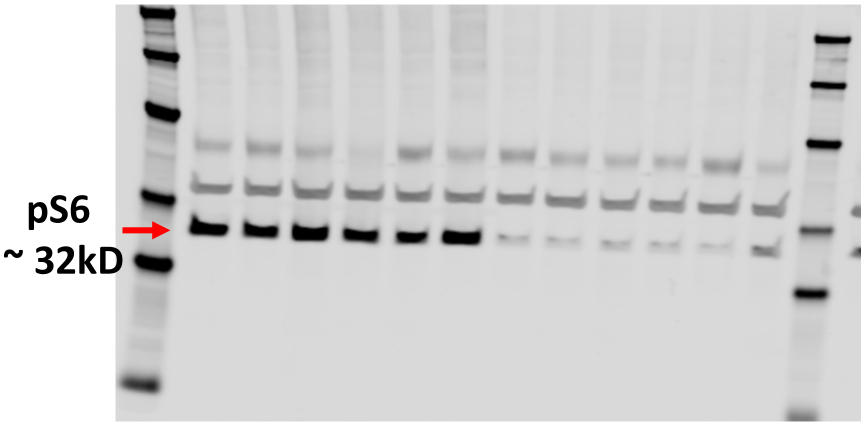

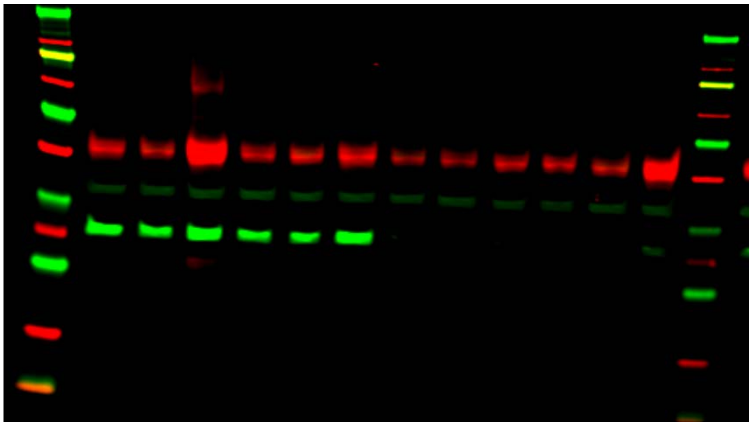

pS6
~ 32kD

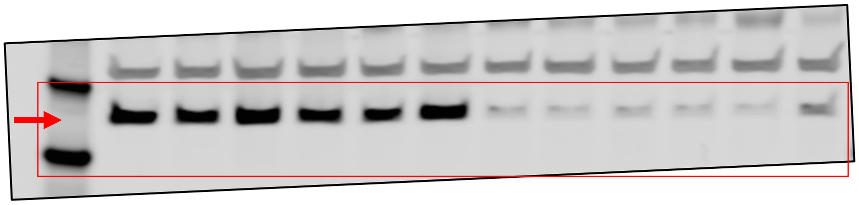

Tubulin
~ 52kD

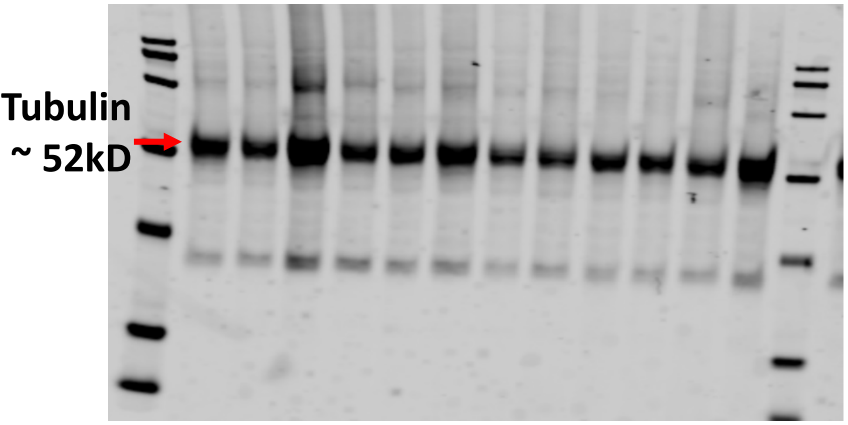

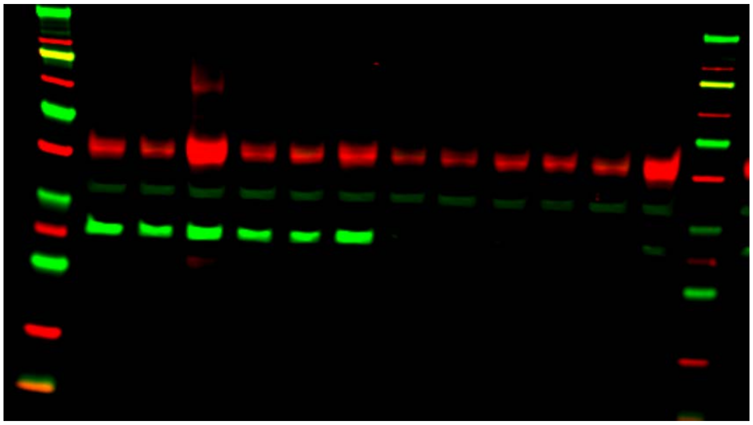

Tubulin
~ 52kD

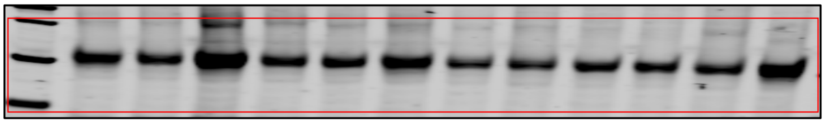

Supplement: S3 Fig — (PDF) [file pone.0251895.s006.pdf]
